# Supplementary material for: Ca2+ signals initiate at immobile IP3 receptors adjacent to ER-plasma membrane junctions
Source: Nat Commun. 2017 Nov 15;8:1505. doi: 10.1038/s41467-017-01644-8 (PMC5686115; doi:10.1038/s41467-017-01644-8)
Supplement: Supplementary file 1 — Supplementary Information [file 41467_2017_1644_MOESM1_ESM.pdf]

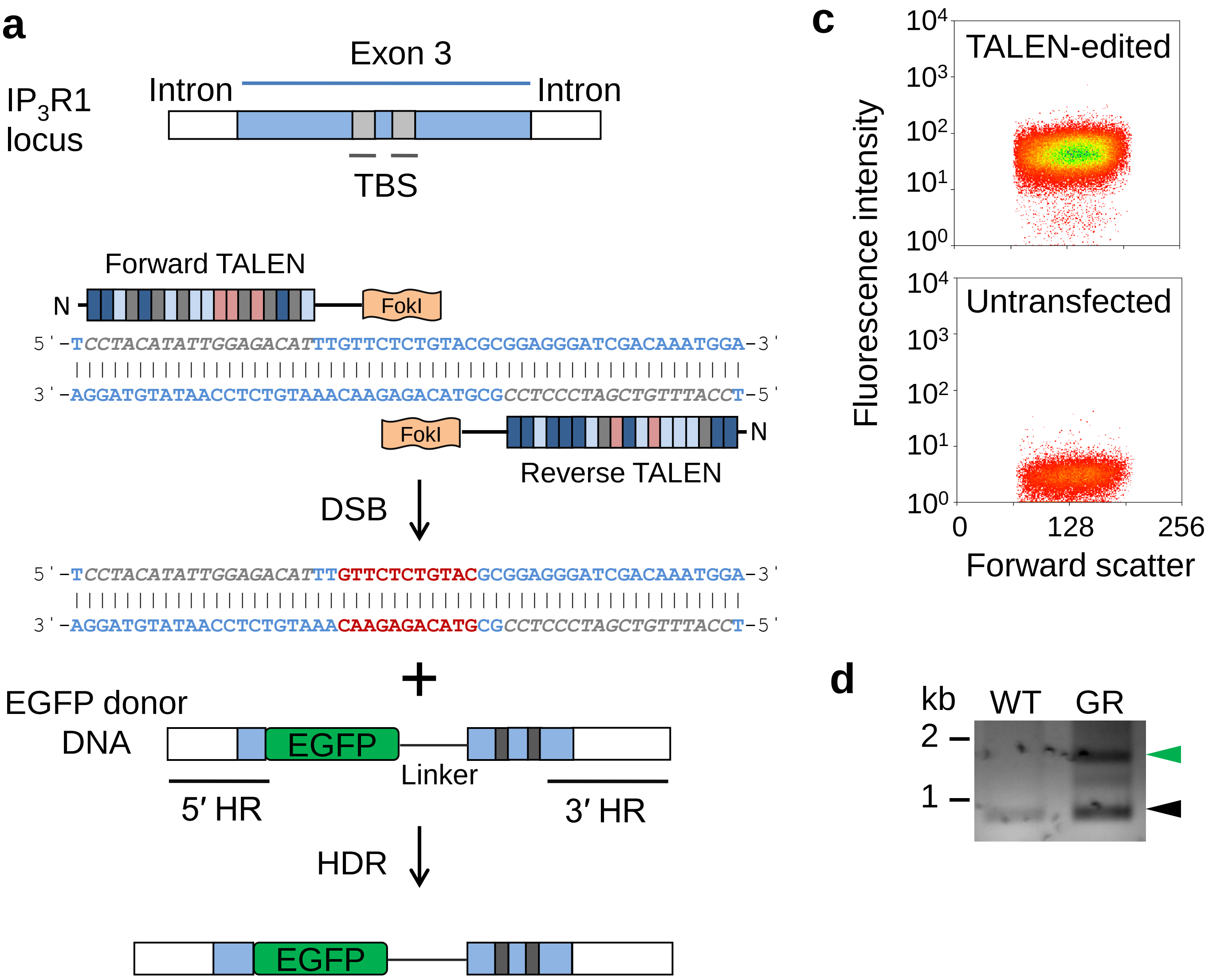


**b**

| TALEN | RVD |
|---|---|
| $IP_3R1$ forward | HD-HD-NG-NI-HD-NI-NG-NI-NG-NG-NN-NN-NI-NN-NI-HD-NI-NG |
| $IP_3R1$ reverse | HD-HD-NI-NG-NG-NG-NN-NG-HD-NN-NI-NG-HD-HD-HD-NG-HD-HD |

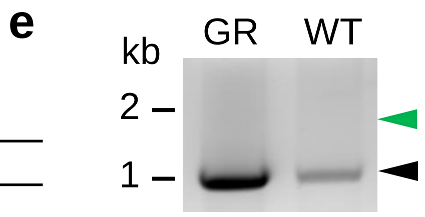


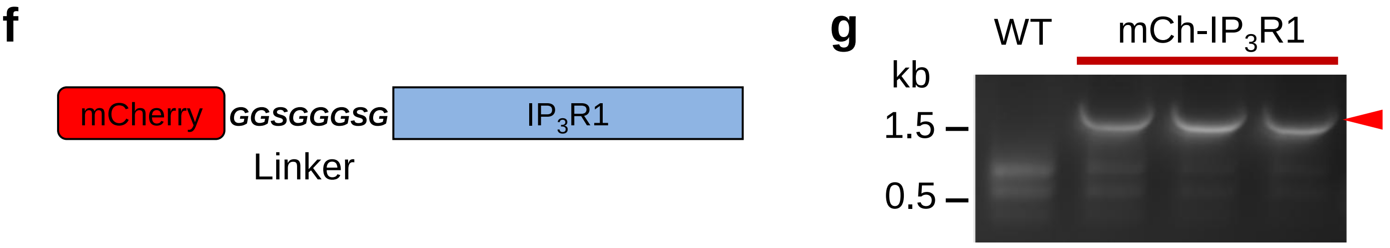


**Supplementary Figure 1 | TALEN-mediated tagging of endogenous $IP_3R1$.**
Legend on next page.

**Supplementary Figure 1 | TALEN-mediated tagging of endogenous $IP_3R1$.** (**a**) Forward and reverse TALENs targeting exon 3 of the $IP_3R1$ gene locus (*itpr1)* allow the FokI nuclease to generate a double-strand break (DSB). In the presence of EGFP donor DNA with its 5' and 3' homology regions (HR), homology-directed repair (HDR) of the break allows insertion of the EGFP coding sequence at the *itpr1* locus. This also incorporates mutations in the TALEN-binding sites (TBS). The original TBS and mutated TBS are shown in light grey and dark grey, respectively. The TBS mutations prevent further TALEN-mediated editing. (**b**) Repeat-variable di-residues (RVD) of the DNA-binding domain of the TALENs used to gene-edit $IP_3R1$. RVDs are the residues that interact with DNA. H, histidine; D, aspartic acid; N, asparagine; G, glycine; I, isoleucine. (**c**) Sequential FACS analysis of TALEN-modified HeLa cells was used to generate cells enriched for expression of EGFP-$IP_3R1$ from which monoclonal cell lines were derived. The FACS analyses are shown for untransfected HeLa cells and the final sorting of EGFP-$IP_3R1$ HeLa cells. (**d**) Genomic DNA from monoclonal EGFP-$IP_3R1$ HeLa cells (GR) and wild-type cells (WT) was PCR-amplified using primers P2F and P4R (see Methods). Bands corresponding to EGFP-$IP_3R1$ (~1.5 kb) and wild-type $IP_3R1$ (~0.8 kb) are indicated by green and black arrows. Positions of standard markers (kb, kilobases) are shown (d, e and g). (**e**) The same genomic DNA was amplified using primers (P13F and P16R) either side of the first codon of the *itpr3* gene, which encodes $IP_3R3$. The native amplicon (black arrow) and the expected position if the EGFP-coding sequence were inserted (green arrow) are shown. The results show that *itpr3* was unmodified in the EGFP-$IP_3R1$ HeLa cells. (**f**) Using TALEN-mediated gene-editing methods similar to those used to tag $IP_3R1$ with EGFP, we tagged endogenous $IP_3R1$ of HEK293 cells with mCherry. Sequencing confirmed the correct insertion of mCherry at exon 3 of *itpr1*. (**g**) Genomic DNA from three monoclonal HEK293 cell lines expressing mCherry-$IP_3R1$ and control HEK293 cells was amplified by PCR using primers mCherry PF and P5R. A PCR product of the expected size for $IP_3R1$ with mCherry inserted (red arrow, 1.6 kb) is present in only the TALEN-modified cell lines.

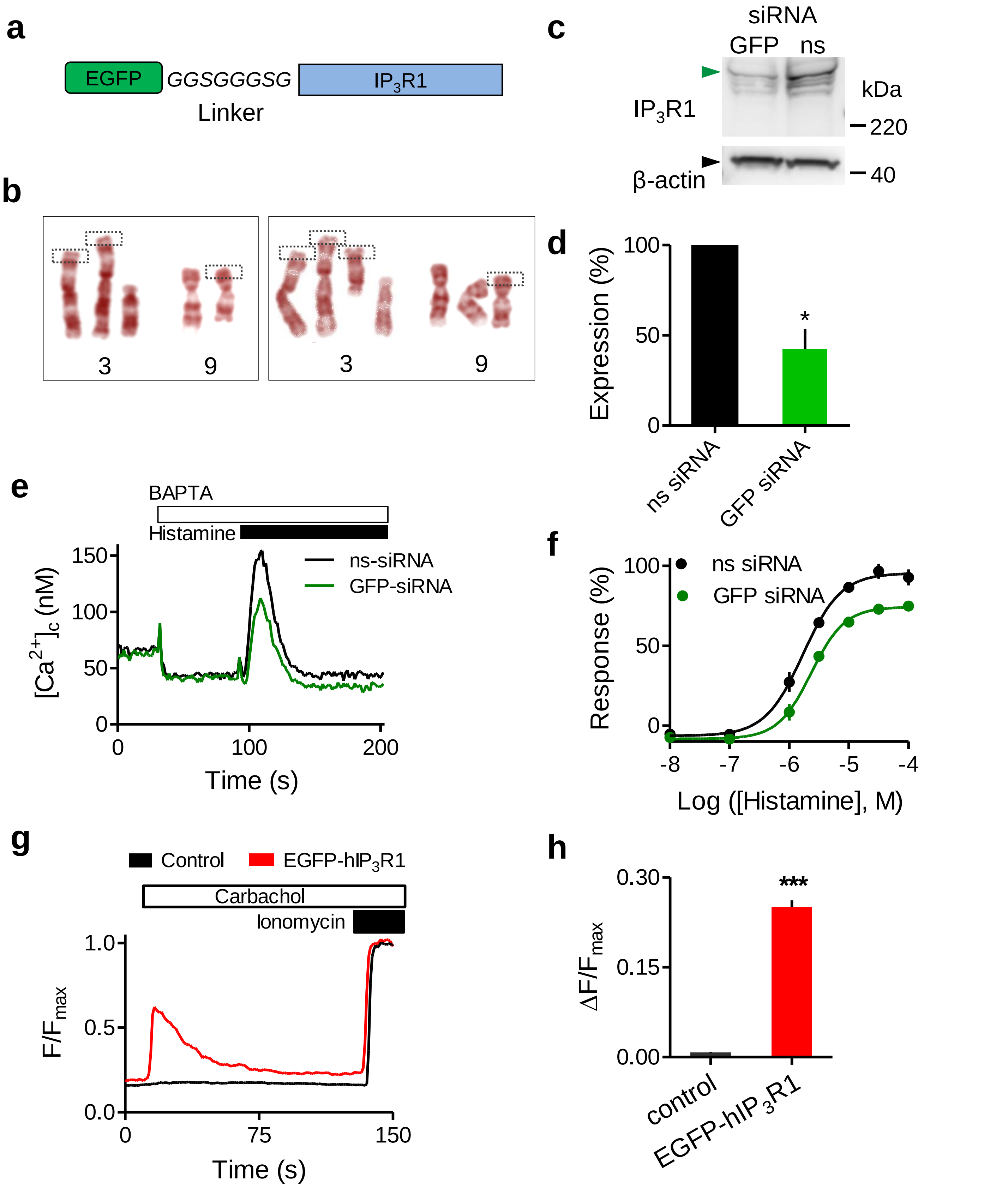


**Supplementary Figure 2 | Endogenous $IP_3R1$s tagged with EGFP form functional channels.** Legend on next page.

**Supplementary Figure 2 | Endogenous $IP_3R1$s tagged with EGFP form functional channels.** (**a**) TALEN-mediated gene-editing was used to attach monomeric EGFP, via a short linker, to endogenous $IP_3R1$ in HeLa cells (**Supplementary Fig. 1**). (**b**) Karyograms of chromosomes 3 and 9 from two cells from the monoclonal EGFP-$IP_3R1$ HeLa cell line used. The 3p26.1 locus (where *itpr1* is located) is boxed, showing 3 copies in one cell and 4 copies in the other, distributed between chromosomes 3 and 9. The heterogenous karyotypes within a monoclonal cell line suggest chromosomal instability, which is well known in HeLa cells[1]. (**c**) WB (using an antiserum to $IP_3R1$) of lysates from EGFP-$IP_3R1$ HeLa cells treated with siRNA against EGFP or a non-silencing (ns) control siRNA. Green arrow indicates the positions of EGFP-$IP_3R1$. (**d**) Summary results show expression of EGFP-$IP_3R1$ after expression of siRNA (%, means ± SEM, n = 3). $^{*}P < 0.05$, Student's *t*-test. (**e**) Populations of EGFP-$IP_3R1$ HeLa cells treated with siRNA against EGFP or ns siRNA were stimulated with histamine (3 µM) after addition of BAPTA (2.5 mM) to chelate extracellular $Ca^{2+}$. Typical traces are shown. (**f**) Summary results (mean ± SEM, n = 4) show the peak increases $[Ca^{2+}]_c$ evoked by histamine after treatment with the indicated siRNAs (as % of the maximal response to histamine in cells treated with ns siRNA). (**g**) HEK cells devoid of endogenous $IP_3R$s (HEK-KO) were transfected to allow transient expression of an EGFP-$IP_3R1$ that replicated that expressed in EGFP-$IP_3R1$ HeLa cells. Cytosolic $Ca^{2+}$ signals evoked by carbachol (1 mM), which stimulates $IP_3$ formation, and ionomycin (5 µM) were detected with the genetically encoded $Ca^{2+}$ sensor, RGECO1[2]. Traces from individual cells show responses as $F/F_{max}$, where $F_{max}$ is the peak fluorescence recorded after addition of ionomycin. (**h**) Summary results show the peak $Ca^{2+}$ signals evoked by carbachol (mean ± SEM, n = 3 fields with 2-11 cells in each), $^{***}P < 0.001$, unpaired Student's *t*-test. Results (c-h) demonstrate that the EGFP-$IP_3R1$s expressed in gene-edited HeLa cells are functional.

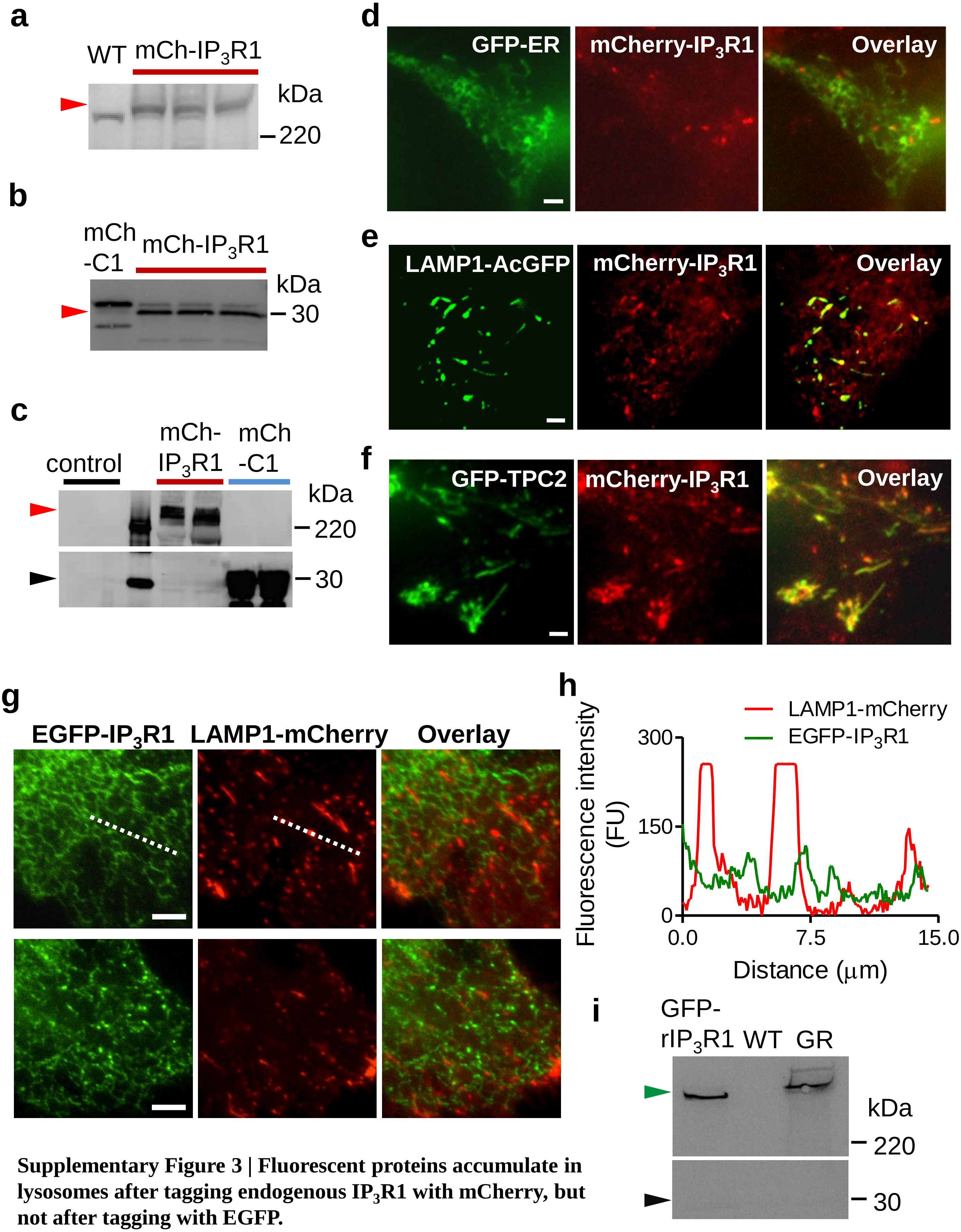


**Supplementary Figure 3 | Fluorescent proteins accumulate in lysosomes after tagging endogenous $IP_3R1$ with mCherry, but not after tagging with EGFP.**
Legend on next page.

**Supplementary Figure 3 | Fluorescent proteins accumulate in lysosomes after tagging endogenous $IP_3R1$ with mCherry, but not after tagging with EGFP.** (**a**) WBs (using an antibody to $IP_3R1$) of lysates prepared from wild-type (WT) HEK293 cells or from three different monoclonal cell lines expressing gene-edited mCherry-$IP_3R1$ (mCh-$IP_3R1$). Similar results were obtained in at least 2 independent analyses, confirming expression of mCh-$IP_3R1$ (~290 kDa, red arrow) in the gene-edited cells. $M_r$ markers (kDa) are shown (a-c and i). (**b**) Supernatants from lysates of three different monoclonal mCherry-$IP_3R1$ HEK293 cells or HEK293 cells transfected to express mCherry-C1 (mCherry with a short additional C-terminal sequence) were immunoprecipitated with anti-RFP beads and equivalent volumes of the pull-downs were analyzed by WB using an anti-mCherry antibody. The WB, which shows only the region corresponding to mCherry (~27 kDa, red arrow), confirms that in mCh-$IP_3R1$ HEK293 cells there is substantial accumulation of free mCherry. Results are from a single experiment. (**c**) Lysates were prepared from FACS-sorted polyclonal HEK293 cells expressing mCh-$IP_3R1$ or wild-type HEK293 cells transiently transfected to express cytosolic mCherry (mCh-C1) or with the empty pcDNA3.1(+) plasmid (control). Equivalent volumes of lysates were immunoprecipitated with anti-RFP beads and the pull-downs were subjected to WB using an anti-mCherry antibody. The results (from a single experiment) demonstrate that mCh-$IP_3R1$ is expressed in the TALEN-modified cells, but this is accompanied by many smaller mCherry-tagged $IP_3R1$ fragments. (**d-f**) TIRFM images of an mCh-$IP_3R1$ HEK293 cell transfected with GFP-ER (d), or with the lysosomal proteins, LAMP1-AcGFP (e) or GFP-TPC2 (f). The overlays show significant co-localization of mCherry with ER (Pearson's coefficient using Costes automatic threshold = 0.718, Costes *P* value = 1.00) and lysosomes (0.567 for LAMP1-AcGFP; 0.857 for GFP-TPC2, Costes *P* value = 1.00). Scale bars (d-f) = 5 µm. The results demonstrate that the endogenous $IP_3R1$ of HEK293 cells was successfully tagged with mCherry, but the cells could not be used for further analysis because mCherry accumulated in lysosomes, where it remained fluorescent. (**g**) TIRFM images of EGFP-$IP_3R1$ in two different EGFP-$IP_3R1$ HeLa cells transfected with LAMP1-mCherry. The overlays show no significant co-localization (Pearson's coefficient with Costes automatic threshold = -0.02 ± 0.02, Costes *P* value = 1.00, n = 3 cells). Scale bars = 5 µm. (**h**) Fluorescence intensity plot along the transect shown in panel g, confirming the lack of co-localization of EGFP ($IP_3R1$) and mCherry (lysosomes). (**i**) Lysates from monoclonal EGFP-$IP_3R1$ HeLa cells (GR), control HeLa cells (WT) or HeLa cells transfected with GFP-rat $IP_3R1$ were immunoprecipitated with anti-GFP beads, and equivalent volumes of the pull-downs were analyzed by WB using anti-GFP antibody. Green and black arrows indicate EGFP-$IP_3R1$ (~290 kDa) and the region corresponding to free EGFP (~27 kDa), respectively. Results are from a single experiment. These results contrast with those where $IP_3R1$ was tagged with mCherry, where there was substantial accumulation of free mCherry (b).

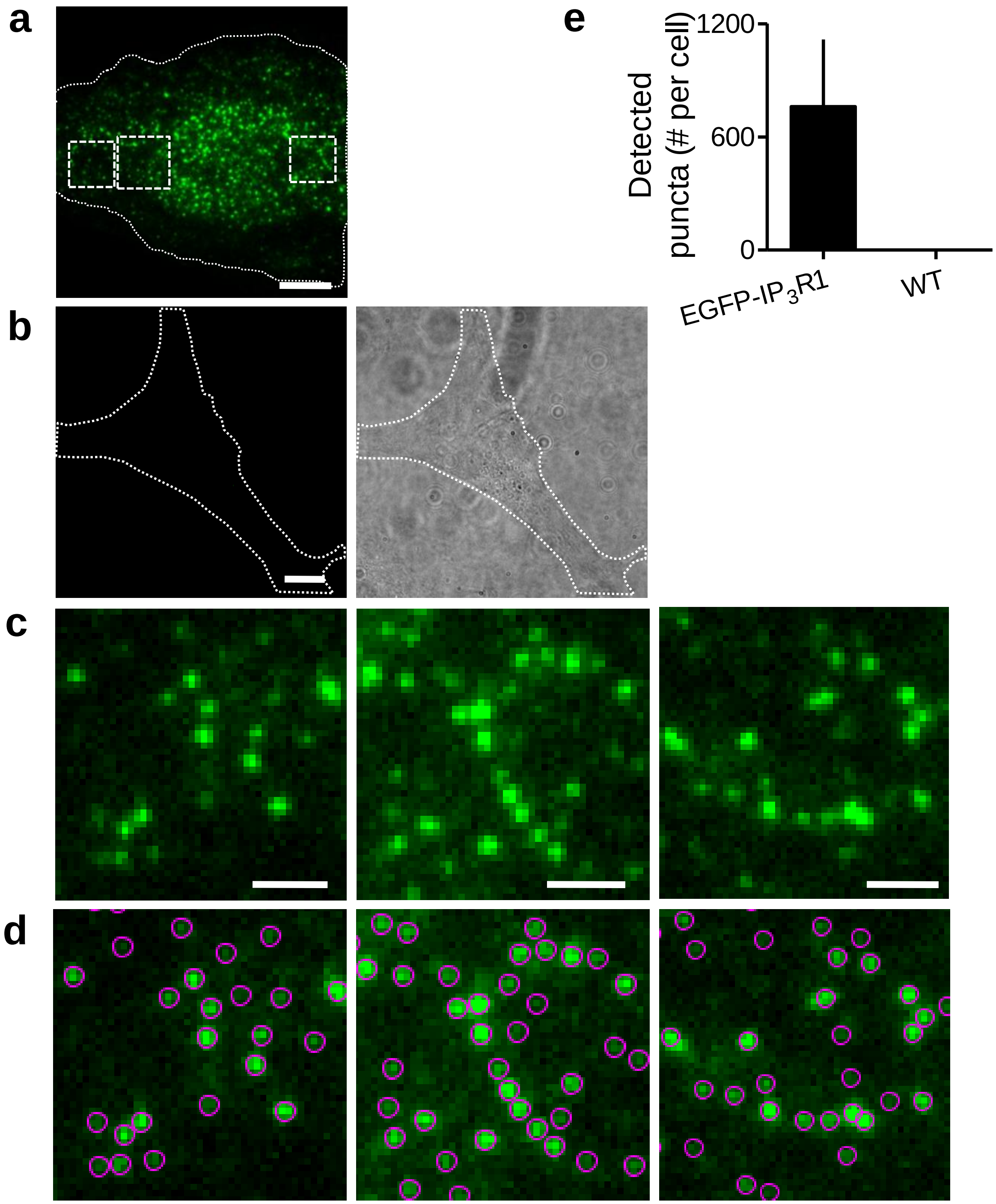


**Supplementary Figure 4 | TrackMate reliably detects EGFP-IP$_3$R1 puncta.** (**a,b**) TIRFM images of an EGFP-IP$_3$R1 HeLa cell (a) and a wild-type (WT) HeLa cell with the cell outlines shown (b) imaged under identical conditions. A brightfield image is shown only for the WT cell. Scale bars = 10 µm. EGFP puncta are observed only in the gene-edited cells. (**c,d**) TrackMate was used to identify EGFP puncta automatically. The images show enlargements of the boxed areas in panel a (c), and the puncta identified by TrackMate (purple circles, d). Scale bars = 2 µm. (**e**) Summary shows the number of puncta detected in TIRFM images of EGFP-IP$_3$R1 and WT HeLa cells (# per cell, mean ± SD, n = 3 cells).

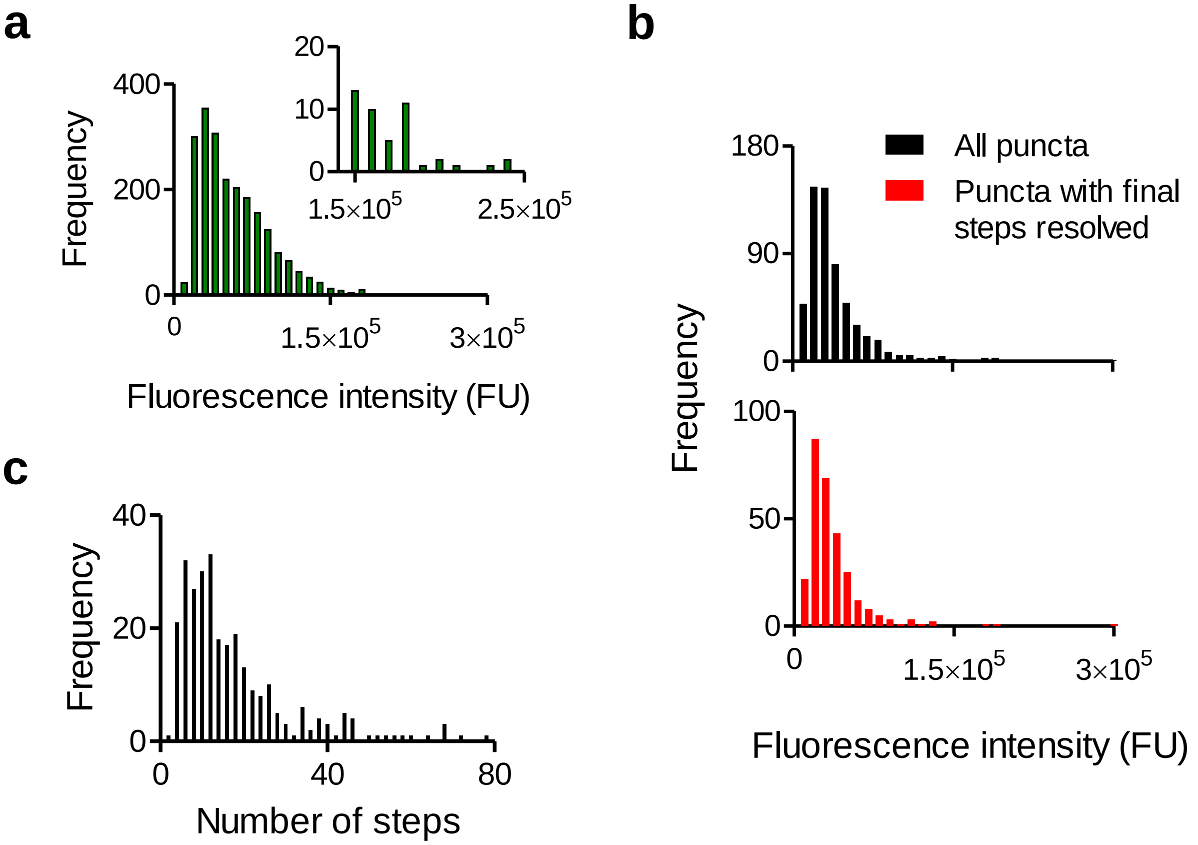


**Supplementary Figure 5 | Analysis of single-step photobleaching.** (**a**) Frequency distribution of the fluorescence intensities of individual puncta in live EGFP-$IP_3R1$ HeLa cells determined using TIRFM. Results (2171 puncta) are from 5 cells. The inset shows the intensity distribution of the most fluorescent puncta on an expanded scale. FU, fluorescence units. A similar analysis of the distribution of fluorescence intensities between mobile and immobile puncta is shown in **Fig. 2f**. (**b**) For photobleaching analyses, puncta were randomly selected from TIRF images of fixed cells. Distributions of fluorescence intensities are shown for all puncta and the puncta accepted for single-step photobleaching analysis (i.e. puncta where the final bleaching step was resolved). From 579 randomly selected puncta in 5 cells, 284 puncta were amenable to analysis. The mean intensity/punctum was 52877 ± 34148 FU/punctum for live cells, and 31698 ± 28828 FU/punctum for puncta accepted for analysis. (**c**) Summary results from the single-step photobleaching analysis show the frequency distribution of the number of fluorophores/punctum (284 puncta from 5 cells), from which the number of tetrameric $IP_3Rs$/punctum was estimated (**Fig. 1f**). Mean number of steps/punctum = 16.5 ± 14 (mean ± SD). To estimate the number of tetrameric $IP_3Rs$/punctum (**Fig. 1f**), we assume all $IP_3R1$ are tagged with EGFP (**Fig. 1c**), fluorescence from EGFP is detected with 80% efficiency[3], $IP_3R1$-3 subunits assemble randomly into tetramers (**Fig. 1d**), and 61% of subunits are $IP_3R1$[4].

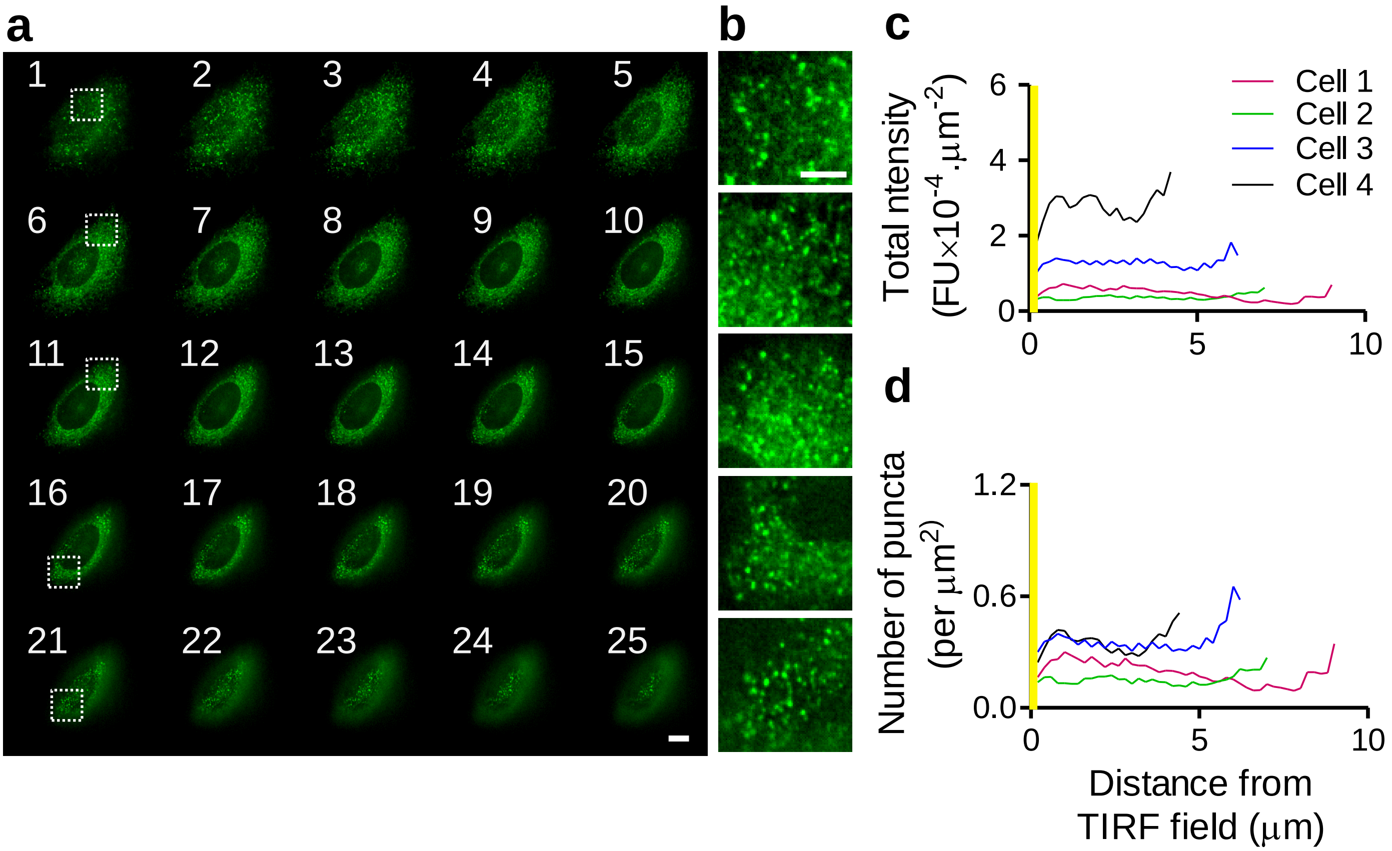


**Supplementary Figure 6 | Distribution of puncta across confocal sections of EGFP-IP$_3$R1 HeLa cells.** (**a**) Spinning disc confocal microscopy with deconvolution was used to generate optical sections across a live EGFP-IP$_3$R1 HeLa cell (200 nm between sections, numbered 1-25 from the image closest to the culture dish). The results show EGFP-IP$_3$R1 puncta in every section, with greater concentrations in perinuclear regions. Scale bar = 10 µm. (**b**) Enlargements of selected sections. Scale bar = 5 µm. (**c,d**) Automated particle detection (Fiji TrackMate) was used to determine the number and intensities of puncta in each z-section across 4 cells. Summary results show the average fluorescence intensity due to puncta (fluorescence units (FU)/µm$^2$) (c) and the number of puncta (per µm$^2$) (d) as a function of distance from the lower surface. The approximate depth of the TIRF field is shown in yellow. The analyses were restricted to sections of sufficient area (>20% of the area detected in image 1) to allow reliable computation of fluorescence densities. The results demonstrate that EGFP-IP$_3$R1 puncta with features similar to those detected by TIRFM are present throughout the cell.

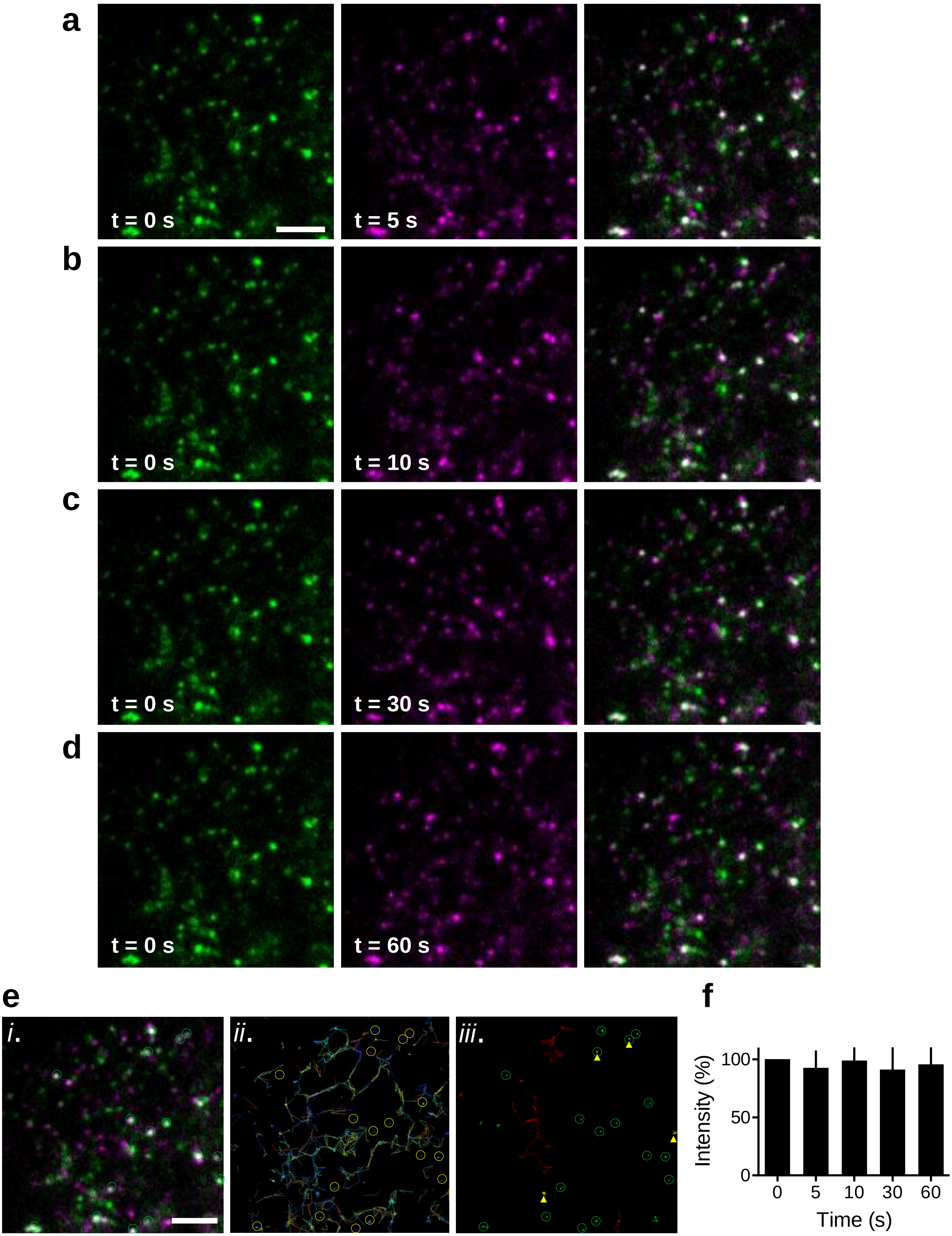


**Supplementary Figure 7 | Identification of immobile puncta.**
Legend on next page.

**Supplementary Figure 7 | Identification of immobile puncta.** To identify immobile puncta, we collected two images of EGFP-IP$_3$R1, pseudocoloured the two images and then overlaid them to identify puncta that had not moved in the intervening period. For most analyses, we collected images at 30-s intervals, but for analyses relating to STORM (**Fig. 4**) we used an interval of 10 s (to minimize bleaching). (**a-d**) Representative TIRFM images of an EGFP-IP$_3$R1 HeLa cell show images collected at intervals of 5 s (a), 10 s (b), 30 s (c), or 60 s (d). Puncta within the first image (t = 0) are pseudocoloured green, and in magenta in the subsequent image. In the overlaid images, immobile puncta appear white. The results demonstrate that images collected at each interval identify the same immobile puncta, and they also confirm that the coincident appearance of different mobile puncta at the same location in successive images does not appreciably contribute to our identification of immobile puncta. Scale bar = 5 µm. (**e**) Comparison of immobile puncta identified using temporal overlays (a-d) or TrackMate with TraJClassifier, which distinguish immobile/sub-diffusive puncta from various forms of mobility according to the distance moved within a defined interval (see **Supplementary Fig. 10**). Green circles in panel *i* (reproduced from c) show every immobile punctum identified using the temporal overlay method (30-s interval). The output from TrackMate shows the trajectories (each in a single colour) of all identified puncta over a period of ~30 s (*ii*). Most puncta are mobile and clearly demarcate the reticular ER, but some show much more restricted movement. The immobile puncta identified in *i* are shown by yellow circles, and they coincide with the puncta with the most restricted mobility. The output from TraJClassifier (*iii*) shows immobile/sub-diffusive puncta as green spots and puncta with confined mobility as yellow spots (highlighted by triangles). Green circles show the immobile puncta identified in panel *i*. The comparison establishes that every particle classified as immobile using the overlay method is also identified as immobile/sub-diffusive or confined when analysed by its single-particle trajectory. Scale bar = 5 µm. (**f**) The fluorescence intensity of each immobile punctum (a-d) was measured at the indicated times and corrected for modest photobleaching by reference to the overall fluorescence intensity of the entire cell. The results (means ± SD, n = 17) confirm that immobile puncta are stable for many minutes.

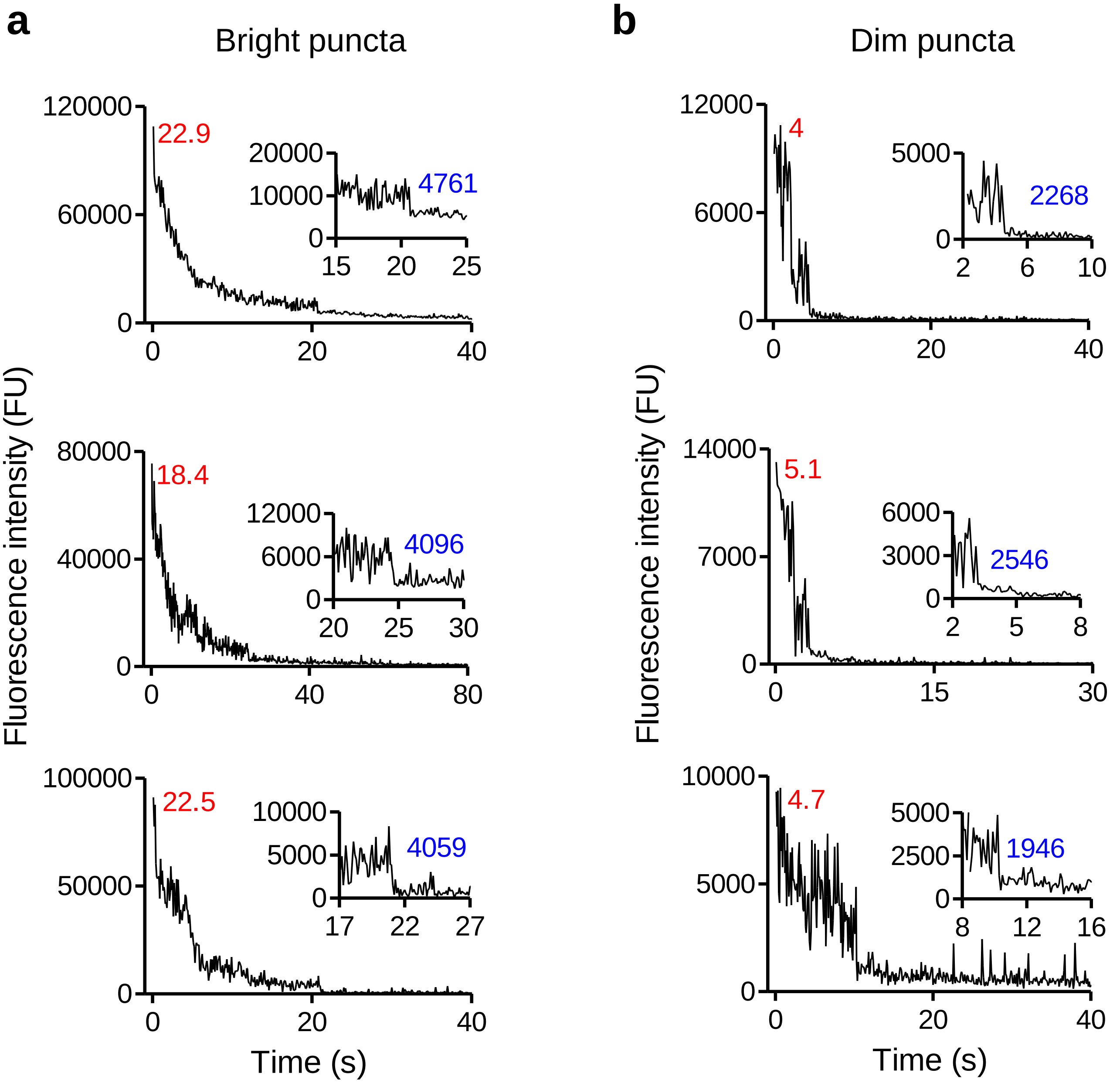


**Supplementary Figure 8 | Single-step photobleaching of bright and dim puncta.** (**a,b**) Representative examples of fluorescence traces used for analyses of single-step photobleaching of the brightest (a) and dimmest (b) puncta. About 80% of the puncta selected were amenable to analysis (i.e. with a resolved final single-step bleaching event). For each trace, the amplitude of the single-step bleaching event is shown on the inset (blue), and the number of fluoroscence steps (red, calculated from the initial fluorescence/amplitude of the single step) is shown in the main panel. The results, showing that the brightest puncta have both larger bleaching steps and more fluorophores, are summarized in **Fig. 2h,i**.

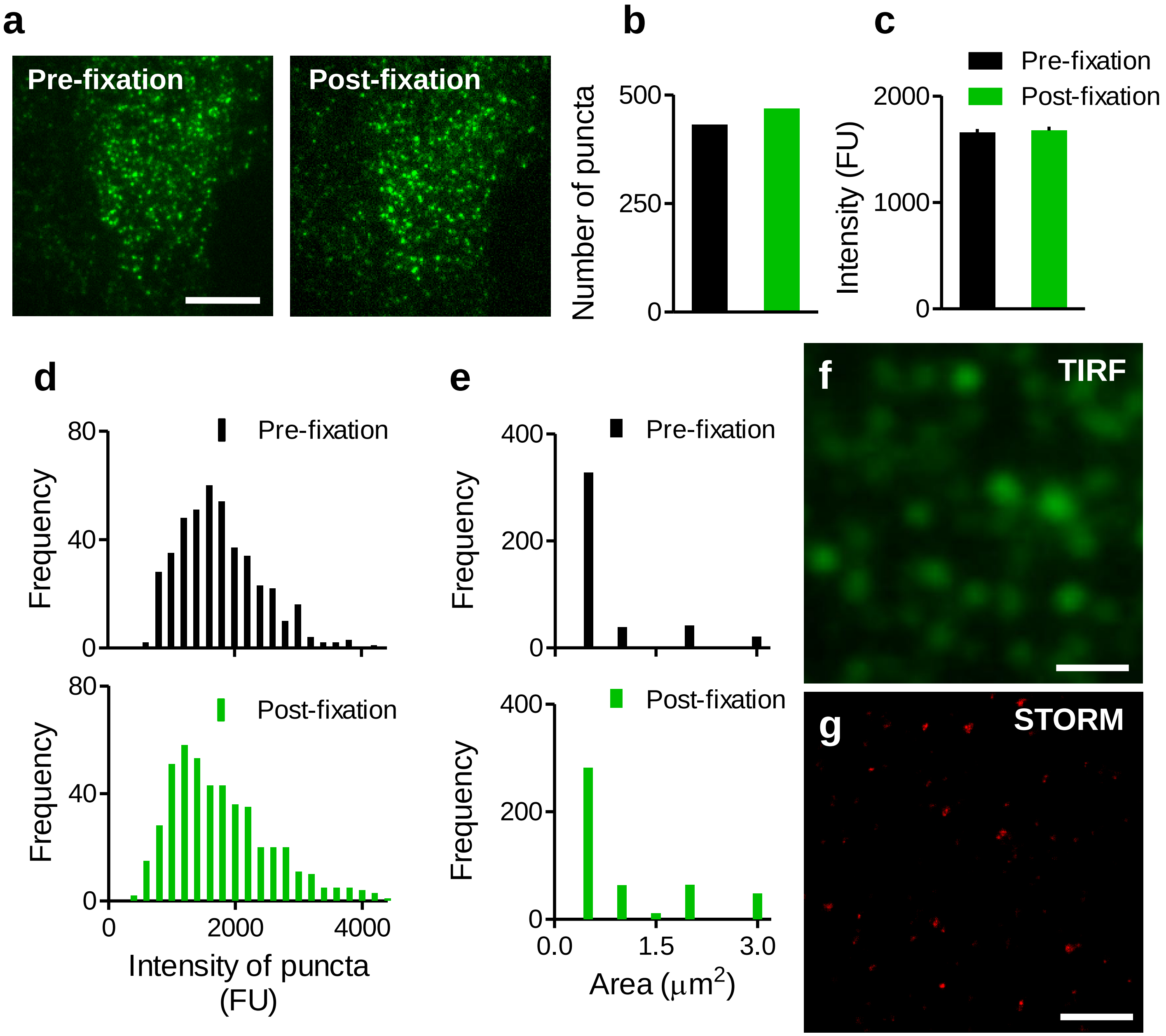


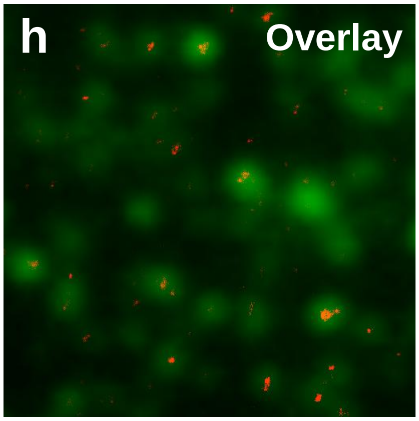


**Supplementary Figure 9 | Fixation for STORM does not perturb the size or distribution of EGFP-IP$_3$R1 puncta.**
(**a**) Representative TIRFM images show the distribution of EGFP-IP$_3$R1 in a single cell before and after fixation for STORM. Scale bar = 10 µm. (**b,c**) Numbers (b, mean) and intensities (c, mean ± SD) of individual EGFP-IP$_3$R1 puncta before and after fixation show no obvious differences. (**d,e**) Distribution of fluorescence intensities of individual puncta (d) and the areas of puncta (e) before and after fixation. FU, fluorescence units. Results (b-e) are from analysis of a single cell (with 432 and 468 puncta analyzed before and after fixation, respectively). (**f-h**) Representative example of a TIRFM image of EGFP-IP$_3$R1 (f), the STORM image (g) and their overlay (h). Scale bars = 1 µm.

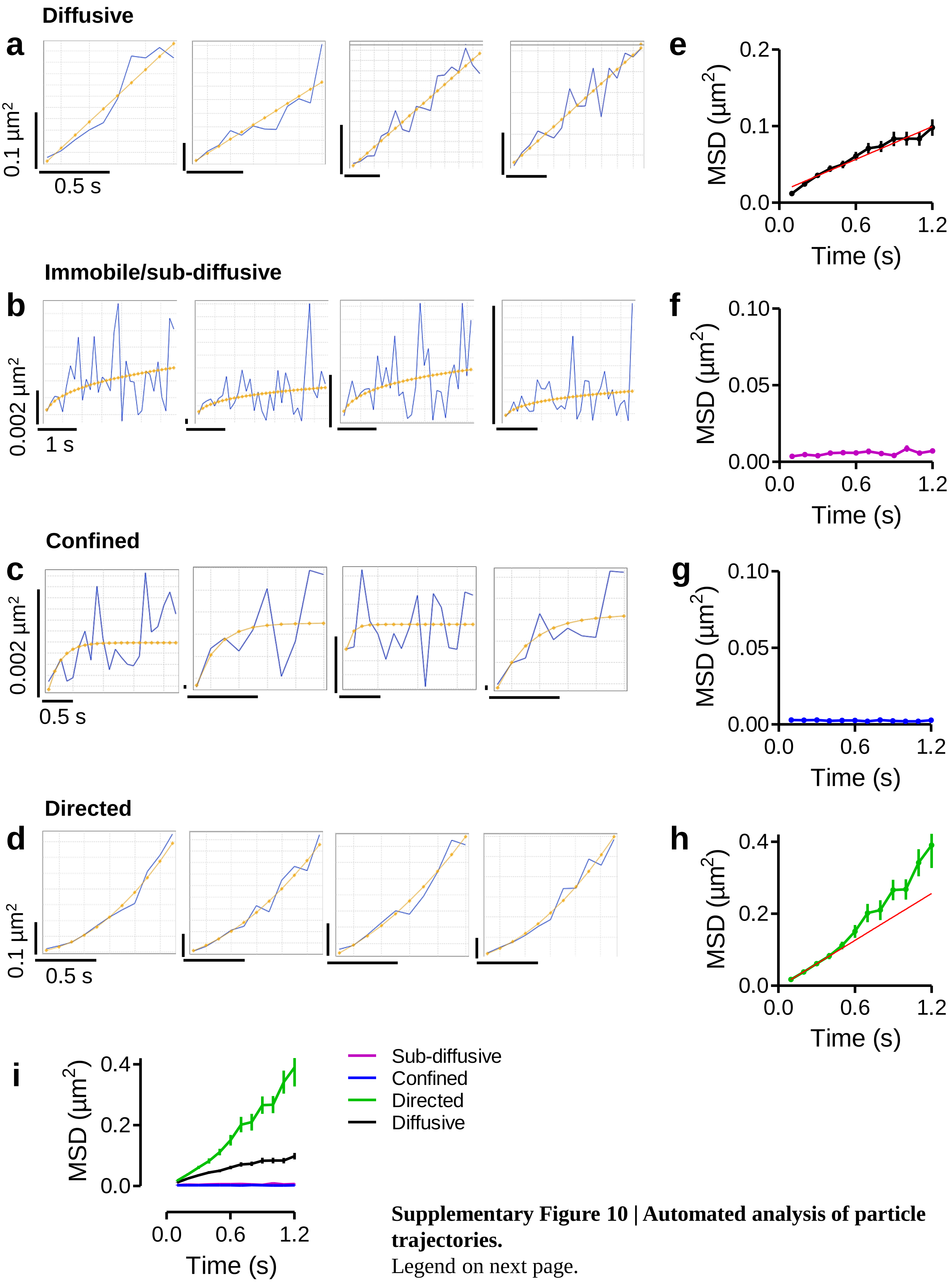


**Supplementary Figure 10 | Automated analysis of particle trajectories.**
Legend on next page.

**Supplementary Figure 10 | Automated analysis of particle trajectories.** (**a-d**) Trajectories of individual EGFP-$IP_3R1$ puncta were recorded at 100-ms intervals for at least 3 s. TraJClassifier allows every non-overlapping interval to provide a measure of mean squared displacement (MSD) for that interval, hence a 3-s recording provides 29 measurements of the MSD for 100 ms, but only 3 measurements for 1 s. Since the algorithm requires a minimum of 3 measurements for each time plotted, a recording of 3 s will provide a time *versus* MSD plot that extends to only 1 s. TraJClassifier uses 9 parameters to categorize individual puncta according to their mobility into diffusive (a); sub-diffusive, most of which are immobile (b); confined (c); and directional (d). For each category, the properties of which are rigorously defined in[5], four representative plots show the relationship between time and MSD for individual puncta (blue) and the curve-fit (orange) from which diffusion coefficients (*D*, a) or speeds (d) were extracted. Time and scale bars (which share the same labels within each group of 4 traces) are shown within each panel. (**e-h**) Summary results show averaged data for 10-50 trajectories for each category. Linear curve-fitting shows (red lines) that the MSD-time plot is linear for diffusive puncta (e), but supralinear for directionally moving puncta (h). The distribution of all puncta between the categories of mobility is summarized in **Fig. 5g**. (**i**) Summary results with data from puncta in all 4 categories of motion plotted on the same scale. From the slope of the MSD-time plot for all diffusive puncta (729 puncta), $D$ for the diffusing particles = $0.0308 \pm 0.002\ \mu m^2.s^{-1}$. The speed of the puncta moving directionally is calculated from the raw distance moved ($0.324 \pm 0.040\ \mu m.s^{-1}$, n = 80). We calculated distances (d) that puncta move during the interleaved recordings (700 ms) of EGFP and $Ca^{2+}$ puffs shown in **Supplementary Fig. 17** from the speed of directionally moving puncta (d = 227 nm), and for diffusing puncta from: $d = \sqrt{2Dt}$, where t = 700 ms (d = 208 nm).

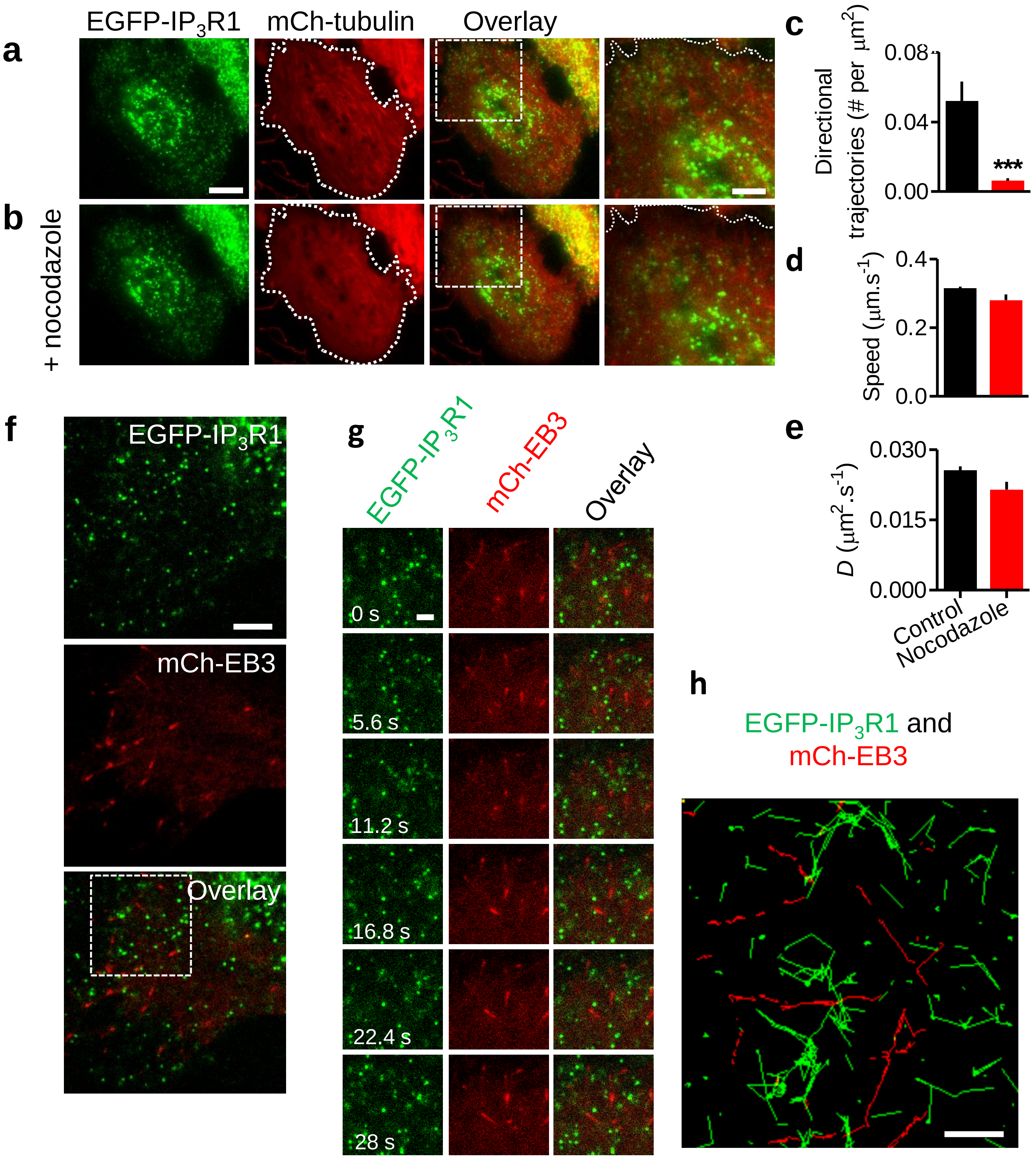


**Supplementary Figure 11 | Directional movement of EGFP-IP$_3$R puncta is not mediated by EB proteins.**
Legend on next page.

**Supplementary Figure 11 | Directional movement of EGFP-IP$_3$R puncta is not mediated by EB proteins.** (**a,b**) Representative TIRF images of an EGFP-IP$_3$R1 HeLa cell expressing mCh-tubulin before (a) and after (b) treatment with nocodazole (10 µM, 30 min). Scale bar = 10 µm (5 µm in enlargements, right panels). Nocodazole causes depolymerization of microtubules and retraction of ER from the cell periphery. (**c-e**) Analyses of trajectories using TraJClassifier show effects of nocodazole on the number of directionally moving IP$_3$R puncta (c), the speed of the directional puncta (d) and $D$ for diffusing puncta (e). $^{***}P < 0.001$, Student's $t$-test relative to control. Results (means ± SEM) are from 355 directional trajectories (c,d) and 3965 diffusive trajectories (e) from 5–11 cells. (**f**) Representative TIRFM images of EGFP-IP$_3$R1 cells expressing mCh-EB3 showing lack of colocalization of EGFP-IP$_3$R1 and mCh-EB3. Scale bar = 5 µm. (**g**) Time series (images shown at 5.6-s intervals) show the distribution of EGFP-IP$_3$R1 and mCh-EB3. Scale bar = 2 µm (**Supplementary Movie 4**). (**h**) The corresponding trajectories (measured over 78 s) of mCh-EB3 (red) and EGFP-IP$_3$R1 (green) show no co-movement. Scale bar = 2 µm.

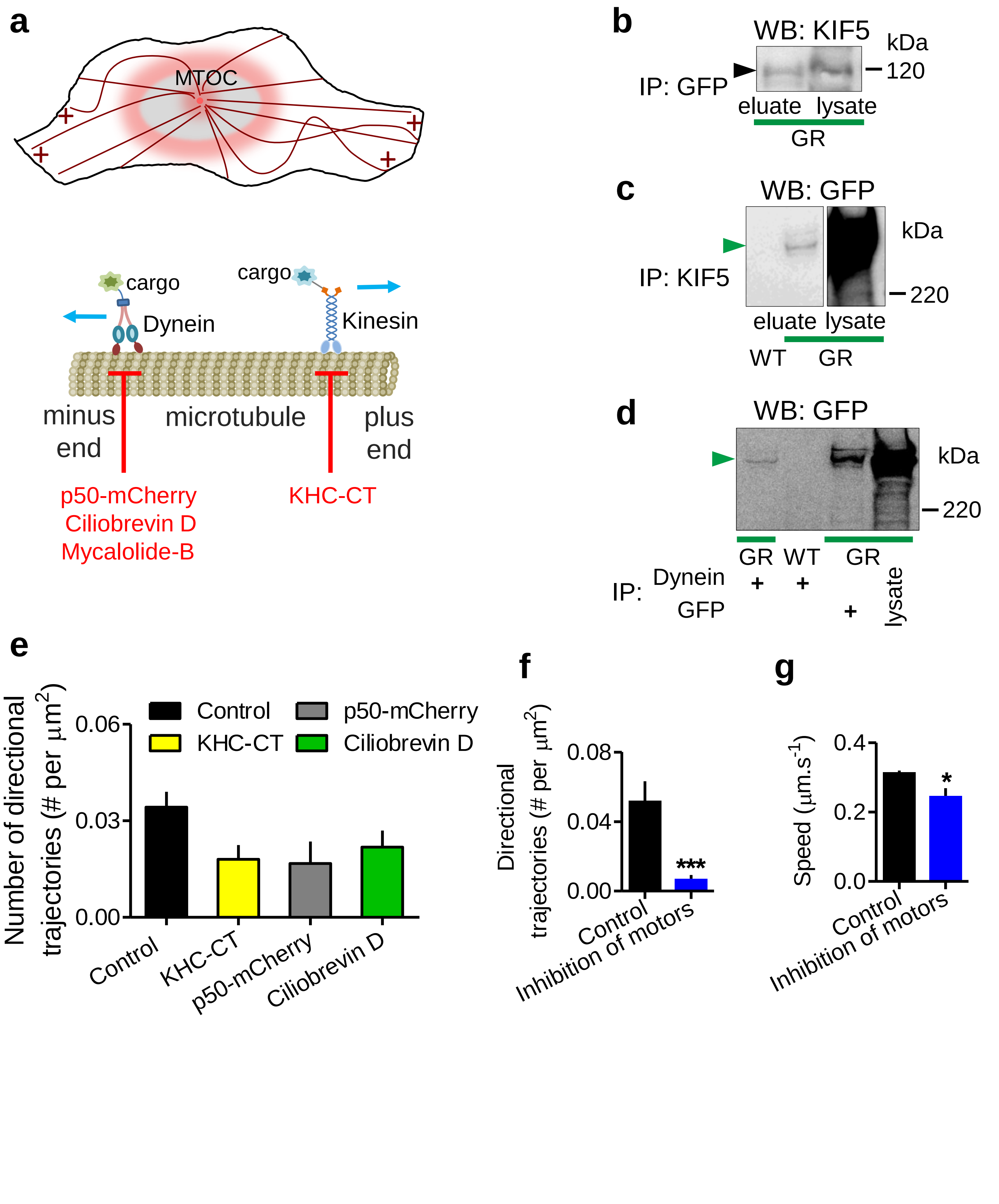


**Supplementary Figure 12 | IP$_3$Rs are moved along microtubules by kinesin and dynein motors.**
Legend on next page.

**Supplementary Figure 12 | $IP_3Rs$ are moved along microtubules by kinesin and dynein motors.** (**a**) Kinesins typically carry cargoes away from the microtubule-organizing centre (MTOC) towards the PM (towards the plus-end of microtubules), and cytoplasmic dyneins move cargoes in the opposite direction (towards the minus-end). Targets of the inhibitors used are shown in red. (**b,c**) WB of lysate from EGFP-$IP_3R1$ HeLa (GR) or wild-type (WT) cells and equivalent amounts of the eluate after immunoprecipitation (IP) with GFP-Trap (WB with KIF5 antibody) (b), or after IP with KIF5 antibody (WB with GFP antibody) (c). Positions of $M_r$ markers (kDa) are shown (b-d). (**d**) Similar analysis after IP with anti-dynein or anti-GFP antibody, and WB with anti-GFP antibody. Results (b-d) are typical of those from two similar experiments. (**e**) Automated tracking analysis (TrackMate and TraJClassifier) of regions of interest (ROI) from the flattest regions of the cell was used to determine the number of directional trajectories of EGFP-$IP_3R1$ puncta after the indicated treatments: control cells (5 cells, 1509 trajectories), expression of a dominant-negative form of kinesin (KHC-CT; 3 cells, 758 trajectories), expression of a dominant-negative form of cytoplasmic dynein (p50-mCherry; 3 cells, 883 trajectories), or treatment with ciliobrevin D (50 µM, 3-12 min; 3 cells, 1342 trajectories). Results show the number of directional trajectories as mean ± SEM (n = 3-5). ANOVA detected no statistically significant differences. (**f,g**) Similar analyses (270 directional trajectories from 5 cells for control; 49 directional trajectories from 7 cells for inhibitors) show the effects of inhibiting both dynein (mycalolide-B, 2 µM, 20 min) and kinesin motors (expression of KHC-CT) on the number of directional trajectories (f) and the speed of the puncta following directional trajectories (g). $^{***}P < 0.001$, $^{*}P < 0.05$, Student's *t*-test relative to control.

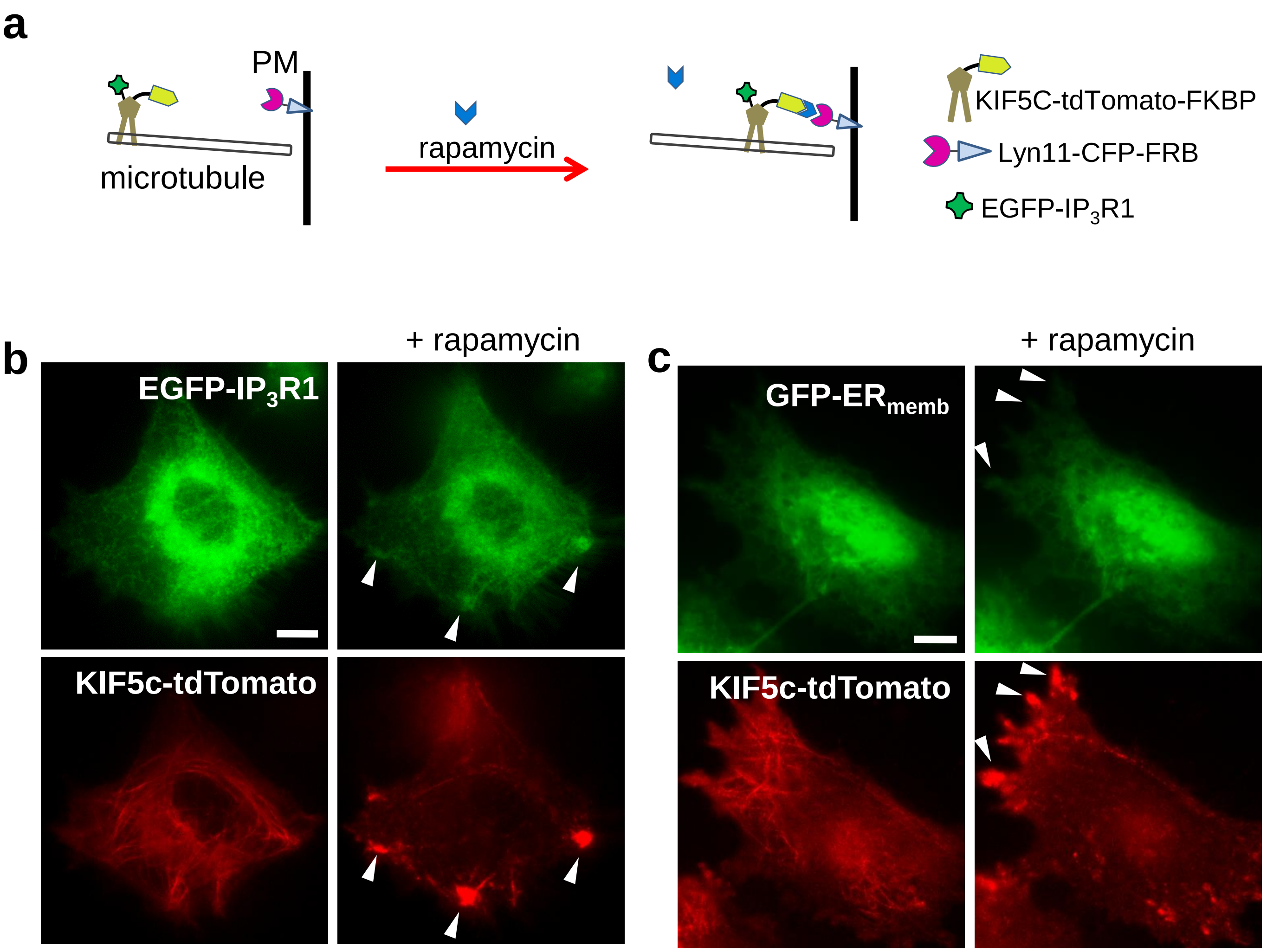


**Supplementary Figure 13 | Kinesin conveys IP$_3$Rs towards the PM.** (**a**) Rapamycin allows dimerization of KIF5C-tdTomato-FKBP (kinesin-1) and FRB targeted to the PM by Lyn11. FKBP, FK506-binding protein; FRB, FKBP/rapamycin-binding domain. (**b,c**) Typical epifluorescence images show an EGFP-IP$_3$R1 HeLa cell expressing KIF5C-tdTomato-FKBP and Lyn11-CFP-FRB (b), or a wild-type HeLa cell expressing the same constructs with GFP-ER$_{memb}$ (c). The distributions of EGFP and tdTomato are shown before and 10-15 min after treatment with rapamycin (1 µM). Dimerization of FKBP and FRB caused kinesin-1 to accumulate at the cell periphery (white arrows) where some EGFP-IP$_3$R1, but not GFP-ER$_{memb}$, also accumulates. Scale bars = 10 µm. In 4 of 6 cells analysed, there was a clear coincident movement of kinesin-1 and EGFP-IP$_3$R1. See **Supplementary Movie 5**.

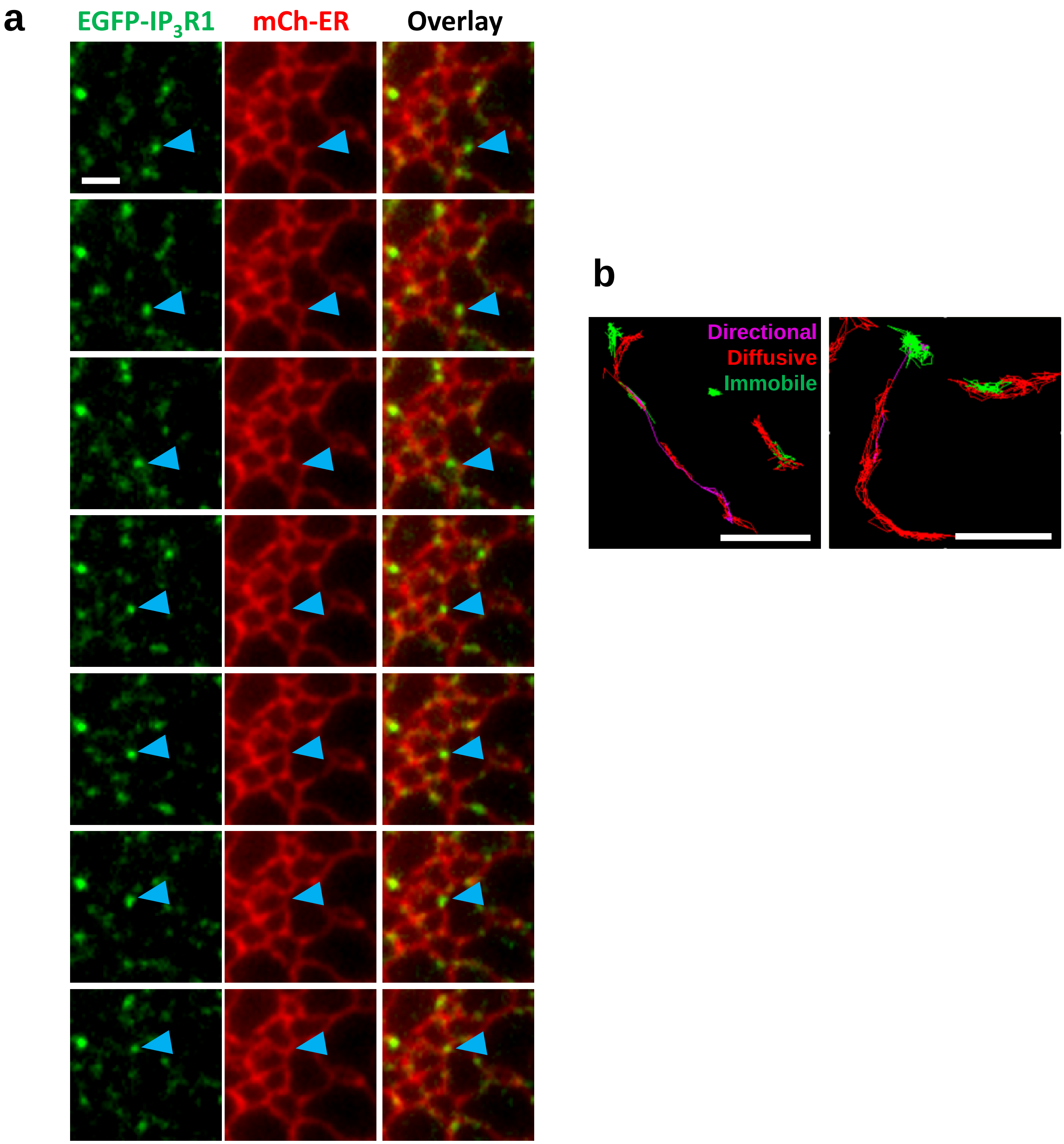


**Supplementary Figure 14 | Mobile $IP_3R$ puncta are unlikely to be within vesicular structures.** (**a**) Time series (0.9 s between images) of TIRFM images from an EGFP-$IP_3R1$ HeLa cell expressing an ER luminal protein, mCh-ER, show no coincident concentration of mCh-ER at a directionally moving EGFP-$IP_3R$ punctum (arrows). Scale bar = 2 µm. See **Supplementary Movie 6**. (**b**) The ER structure was defined by recording trajectories of diffusing EGFP-$IP_3R1$ puncta (red), and the trajectories of puncta moving directionally were then superimposed (purple). Immobile puncta are shown in green. Results from 2 cells are shown. Scale bars = 2 µm. These analyses (a, b) suggest that directionally moving $IP_3R$ puncta move within the ER, rather than within separate vesicles.

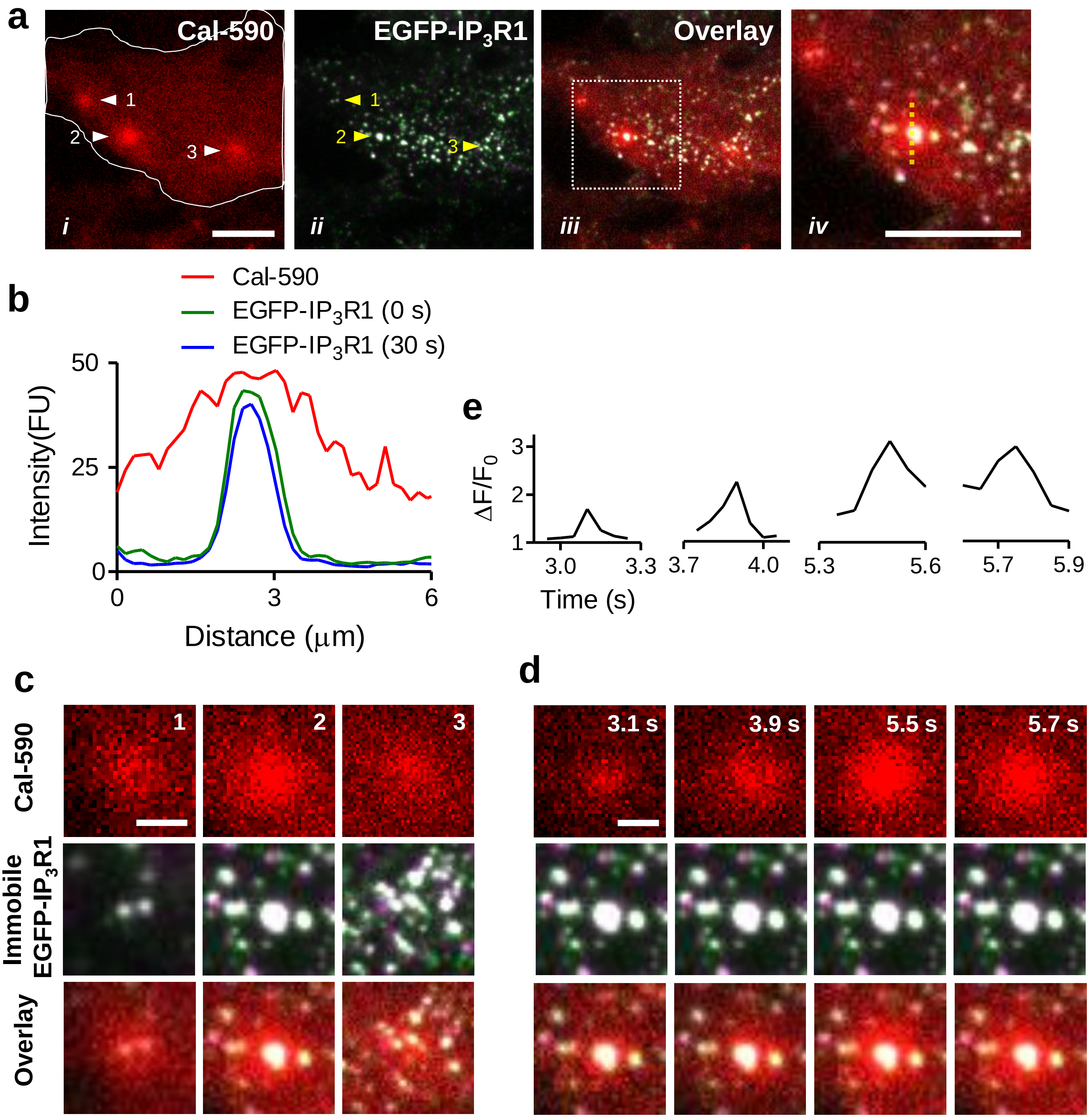


**Supplementary Figure 15 | $Ca^{2+}$ puffs evoked by histamine occur at immobile $IP_3Rs$. (a)** TIRFM image of a single EGFP-$IP_3R1$ HeLa cell loaded with EGTA and Cal-590, showing $Ca^{2+}$ puffs evoked by histamine (10 µM) and immobile $IP_3Rs$ (white spots, see **Supplementary Fig. 7**). The boxed area of the overlay image (*iii*) is shown enlarged in (*iv*). Scale bars = 10 µm. (**b**) Colocalization of $Ca^{2+}$ release and an immobile $IP_3R$ punctum shown by their fluorescence intensity profiles along the dashed blue line in a*iv*. The EGFP profiles were captured immediately before and after recording Cal-590 fluorescence (for 30 s). (**c**) Enlargements of the three puff sites highlighted in panel a show the colocalization of each $Ca^{2+}$ release event with an immobile $IP_3R$ punctum. (**d**) Enlargements of site 2 (from a) show four successive puffs generated at the same immobile punctum. Scale bars = 2 µm (c and d). (**e**) The temporal profiles of the Cal-590 fluorescence changes ($\Delta F/F_0$) show that the $Ca^{2+}$ puffs evoked by histamine last ~200 ms.

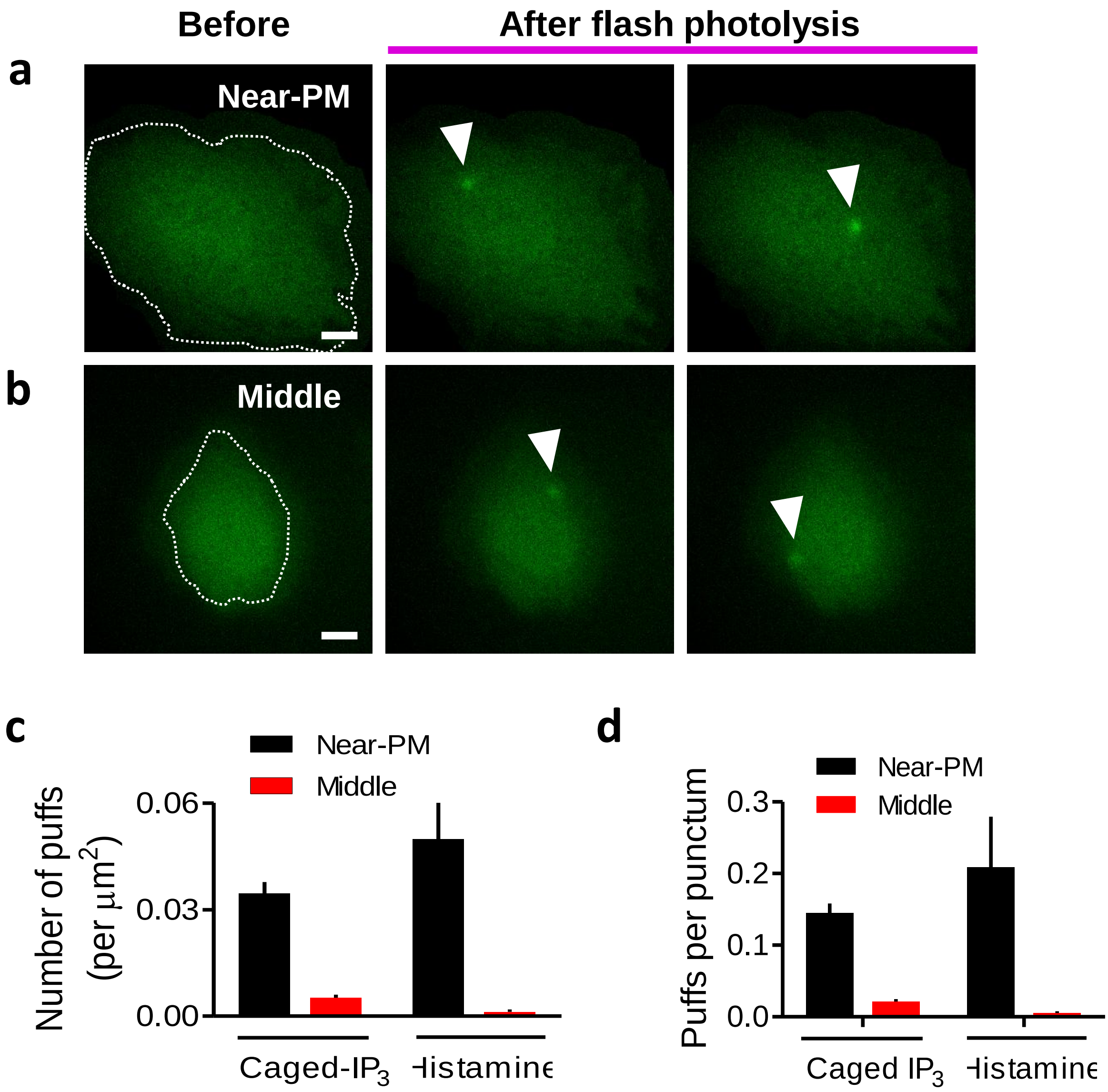


**Supplementary Figure 16 | $Ca^{2+}$ puffs evoked by histamine or photolysis of caged-$IP_3$ occur close to the PM.** (**a,b**) Representative confocal sections from a Cal-520-loaded WT HeLa cell in a plane close to the basal PM (a) and across the middle of the cell (b) showing $Ca^{2+}$ puffs evoked by photolysis of caged-$IP_3$. Scale bars = 10 μm. The rare $Ca^{2+}$ puffs detected at the middle of the cell were invariably close to the PM. (**c**) Summary results (mean ± SEM, n = 3 cells) show the number of puffs detected within each confocal plane for the 30 s after stimulation with histamine (1 μM) or a photolysis flash (25 ms) to photolyse caged-$IP_3$. (**d**) From equivalent confocal sections of puncta in EGFP-$IP_3$R1 cells (**Supplementary Fig. 6)** and of $Ca^{2+}$ puffs in WT HeLa cells (panels a and b), the number of puffs/punctum was estimated. Results are mean ± SEM, n = 3 cells.

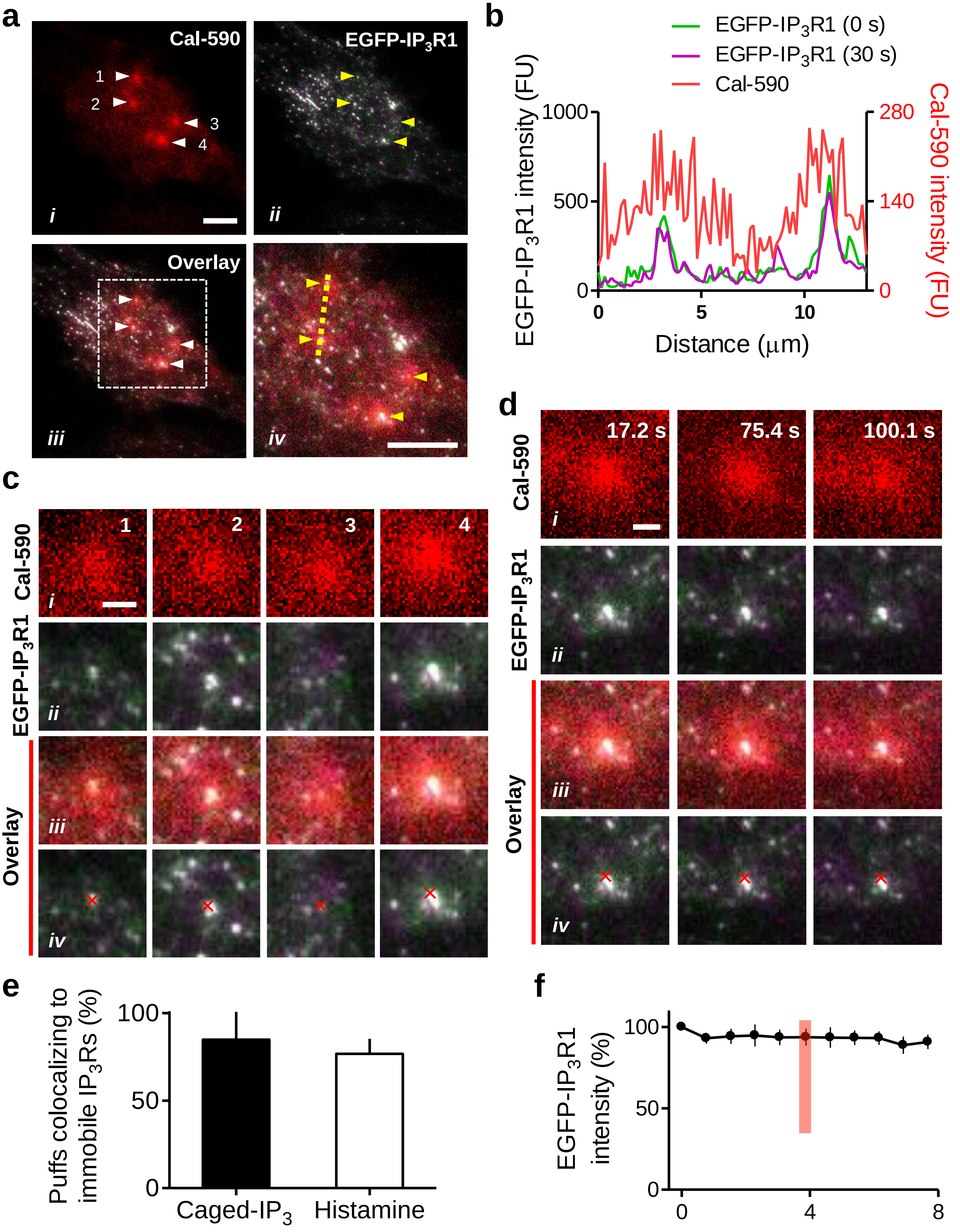


**Supplementary Figure 17 | $Ca^{2+}$ puffs occur at immobile IP$_3$Rs.**
Legend on next page.

**Supplementary Figure 17 | $Ca^{2+}$ puffs occur at immobile $IP_3Rs$.** For these analyses, interleaved images of Cal-590 (561-nm excitation) and EGFP (488 nm) fluorescence were captured, with intervals of ~400 ms between each wavelength (700 ms between successive measurements at the same wavelength). This sequence allowed the relationship between the distribution of $IP_3Rs$ (mobile and immobile) and $Ca^{2+}$ signals to be defined. This method contrasts with that in **Fig. 6** and **Supplementary Fig. 15**, where the locations of EGFP-$IP_3R1$ were determined immediately before and after the 30-s recording of $Ca^{2+}$ puffs. (**a**) Cells loaded with EGTA and Cal-590, were stimulated with histamine (10 µM). TIRFM images show the $Ca^{2+}$ puffs (*i*, arrows) and EGFP-$IP_3R1s$ (*ii*-*iv*) for a single cell. The distribution of EGFP-$IP_3R1s$ is shown 30 s before (green) and immediately after (magenta) the corresponding $Ca^{2+}$ signal. The pseudocoloured overlay images show the distribution of immobile $IP_3Rs$ (white spots, see **Supplementary Fig. 7**). The boxed area of the overlay image (*iii*) is shown enlarged in (*iv*). Scale bars = 10 µm. (**b**) Colocalization of $Ca^{2+}$ release sites and immobile $IP_3R$ puncta (coincident green and magenta) shown by their fluorescence intensity profiles along the dashed line in a*iv*. (**c**) Enlargements of the 4 puff sites highlighted in panel a show colocalization of each $Ca^{2+}$ release event with an immobile $IP_3R$ punctum (*iii*). Overlays (*iv*) of the centres of mass of each $Ca^{2+}$ puff (red cross) and EGFP-$IP_3R1$. (**d**) Similar presentation of enlargements of site 4 (from a) showing three successive puffs generated at the same immobile punctum. Scale bars = 2 µm (c and d). (**e**) Summary shows the fraction of $Ca^{2+}$ puffs evoked by histamine (n = 5 cells, 70 puffs, mean ± SD) or photorelease of $IP_3$ (n = 6 cells, 108 puffs) that occurred at immobile $IP_3R$ puncta. $Ca^{2+}$ puffs and $IP_3R$ puncta were considered to occur at the same site if the centre of mass of the peak change in Cal-590 fluorescence was within 0.96 µm of the punctum. (**f**) For each of the $Ca^{2+}$ puffs shown in panels c and d, the fluorescence intensity of the associated EGFP-$IP_3R1$ punctum was recorded for 5 frames before, and 5 frames after the $Ca^{2+}$ puff. Summary results (mean ± SEM, n = 6) show the fluorescence intensity for each punctum as a percentage of that recorded in the first frame; the interval wherein the $Ca^{2+}$ puff occurred is shown by the red bar.

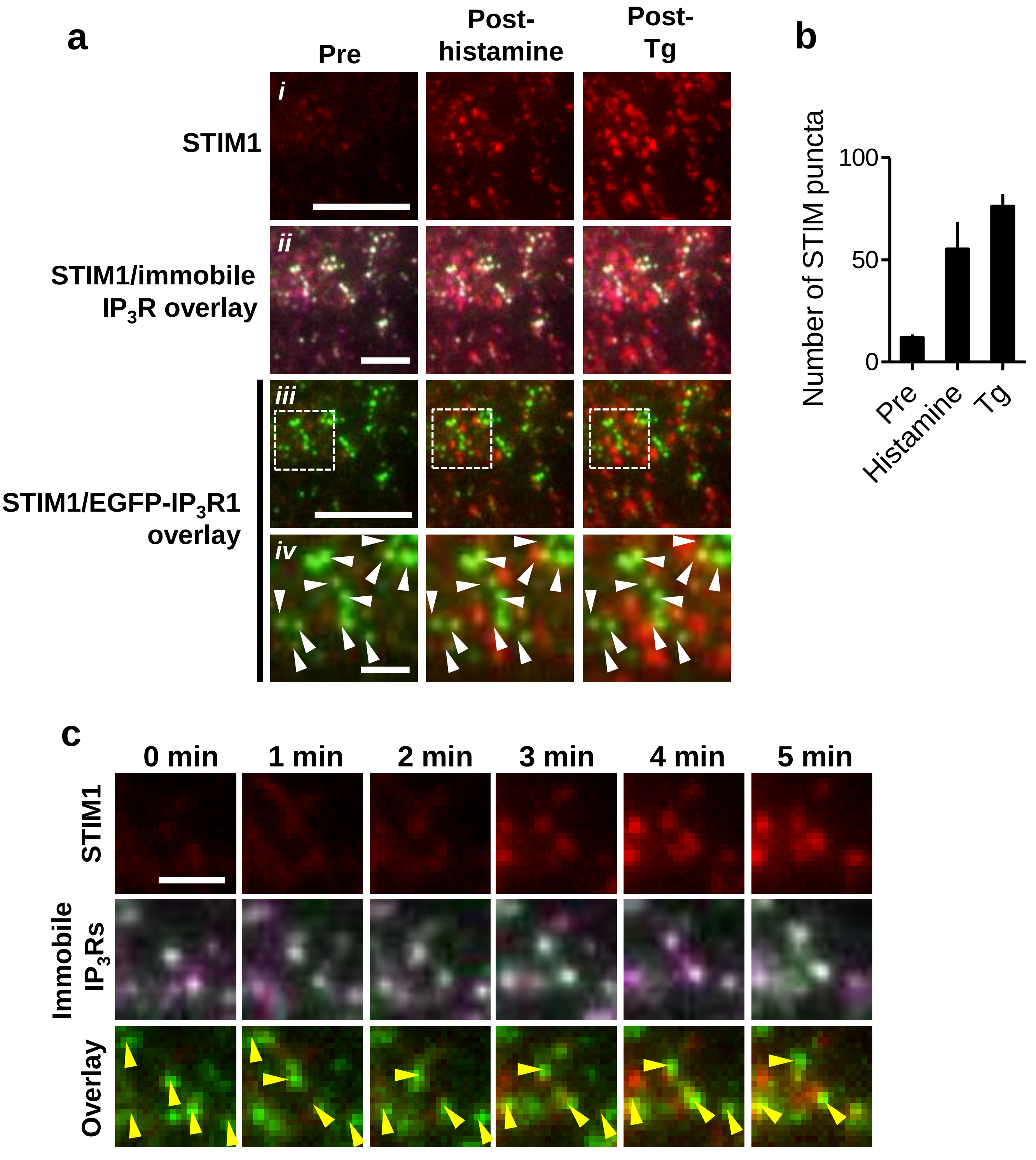


**Supplementary Figure 18 | Histamine causes translocation of STIM1 to ER-PM junctions adjacent to immobile $IP_3R$ puncta.**

Legend on next page.

**Supplementary Figure 18 | Histamine causes translocation of STIM1 to ER-PM junctions adjacent to immobile $IP_3R$ puncta.** (**a**) EGFP-$IP_3R1$ HeLa Cells expressing CFP-STIM1 were stimulated with histamine (10 µM, 30 s). The TIRFM image, recorded immediately afterwards, shows formation of CFP-STIM1 puncta (pseudocoloured in red) at ER-PM junctions. Subsequent addition of thapsigargin (Tg, 1 µM, 15 min) caused formation of additional puncta and enlargement of those formed after stimulation with histamine (*i*). Overlaid images show STIM1 puncta (red) adjacent to immobile $IP_3Rs$ (white) (*ii*). The same images are shown in *iii*, but with $IP_3Rs$ shown in green. In the enlarged images (boxes in *iii*), all immobile $IP_3R$ puncta are shown by arrows (*iv*). Scale bars = 10 µm (*i*-*iii*) or 2 µm (*iv*). (**b**) Summary results (means ± SEM, n = 3 cells) show the number of STIM1 puncta formed before and after stimulation with histamine (10 µM, 30 s) and then thapsigargin (Tg, 1 µM, 15 min). (**c**) Time-lapse TIRFM images show the effects of thapsigargin (1 µM) on the distribution of STIM1-mCherry (red), immobile $IP_3Rs$ (white) and the overlay of STIM1 with EGFP-$IP_3R1$. Arrows in the bottom panels show all immobile $IP_3R$ puncta. We note that the positions of immobile $IP_3R$ puncta need not be exactly coincident across prolonged time series because their immobility is established over a 30-s interval, while the ER may move over longer periods (**Supplementary Fig. 7**). Scale bar = 2 µm.

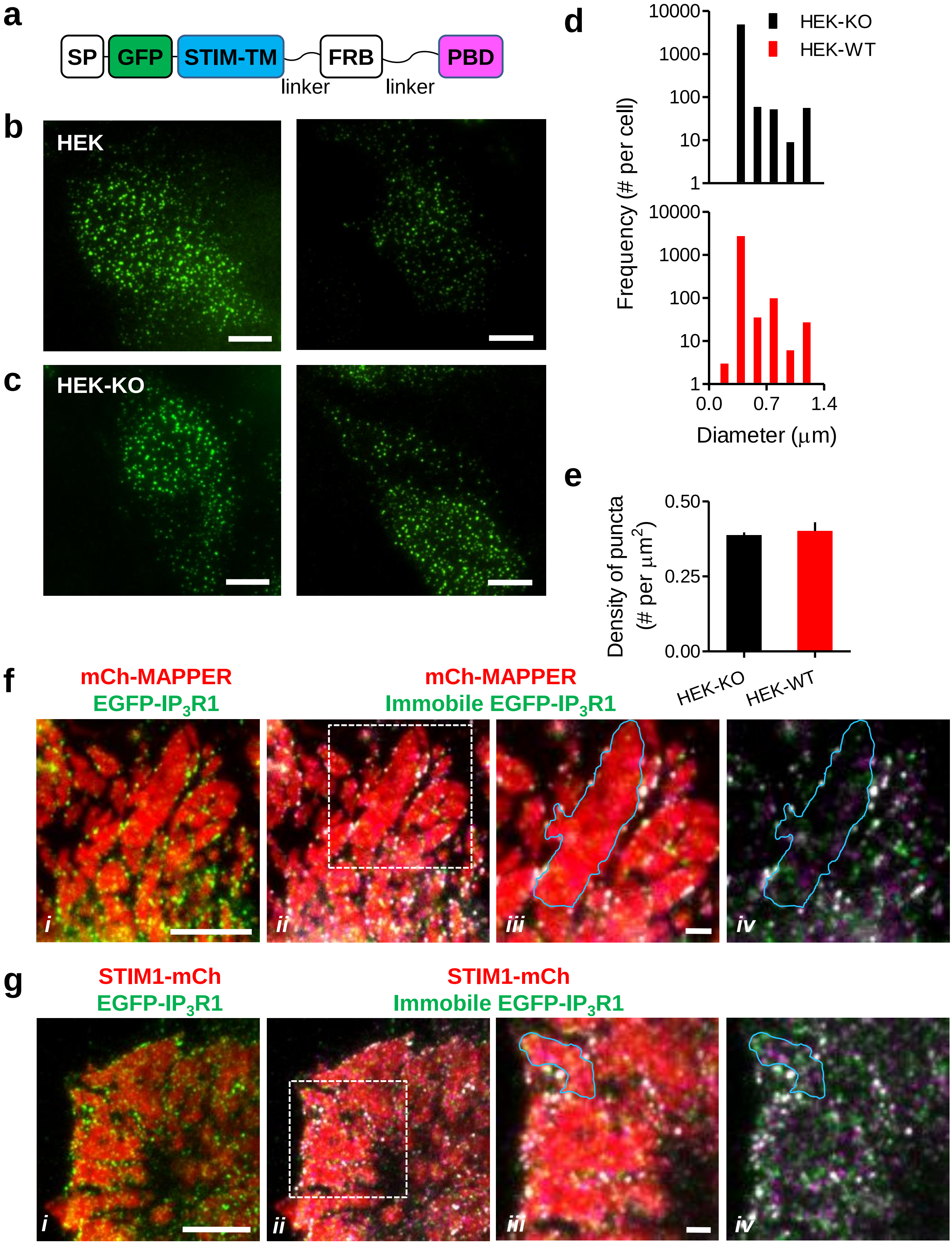


**Supplementary Figure 19 | $IP_3$Rs are not required for assembly of ER-PM junctions.**
Legend on next page.

**Supplementary Figure 19 | $IP_3$Rs are not required for assembly of ER-PM junctions.** (**a**) Structure of GFP-MAPPER[6]. SP, signal peptide; STIM-TM, STIM1 transmembrane domain; FRB, FKBP12-rapamycin binding sequence; PBD, polybasic domain of STIM1. (**b,c**) Representative TIRFM images show expression of GFP-MAPPER, to identify ER-PM junctions, in normal HEK 293 cells (b) and HEK 293 cells without endogenous $IP_3$Rs (HEK-KO cells)[7] (c). Scale bars = 10 µm. (**d,e**) Summary results (mean ± SEM, from 12 cells) show the size (d) and density (e) of GFP-MAPPER puncta. (**f**) Whereas GFP-MAPPER does not perturb ER-PM junctions[6], mCherry-MAPPER exaggerates them (Jen Liou, UT Southwestern Medical Center, personal communication). Representative image of part of an EGFP-$IP_3$R1 HeLa cell expressing mCherry-MAPPER shows exaggerated ER-PM junctions and displacement of immobile $IP_3$Rs to their margins (see **Supplementary Movie 9**). All EGFP-$IP_3$R1 puncta are shown in panel *i* (green), while immobile (white) and mobile (green and magenta) puncta are distinguished in panel *ii*, the enlarged panel (*iii*) and in panel (*iv*). Comparison of the border of an enlarged junction (cyan in *iii* and *iv*) with its enclosed area shows that immobile puncta (white) populate the border, while mobile puncta (magenta and green) populate the enclosed area. (**g**) Representative image of EGFP-$IP_3$R1 HeLa cell massively over-expressing mCh-STIM1, with EGFP-$IP_3$R1 puncta shown as in panel f. Scales bars (f,g) = 10 µm (*i, ii*) and 2 µm (*iii, iv*). These results (b-g) suggest the assembly of ER-PM junctions defines the distribution of immobile $IP_3$R puncta, rather than $IP_3$Rs causing assembly of junctions.

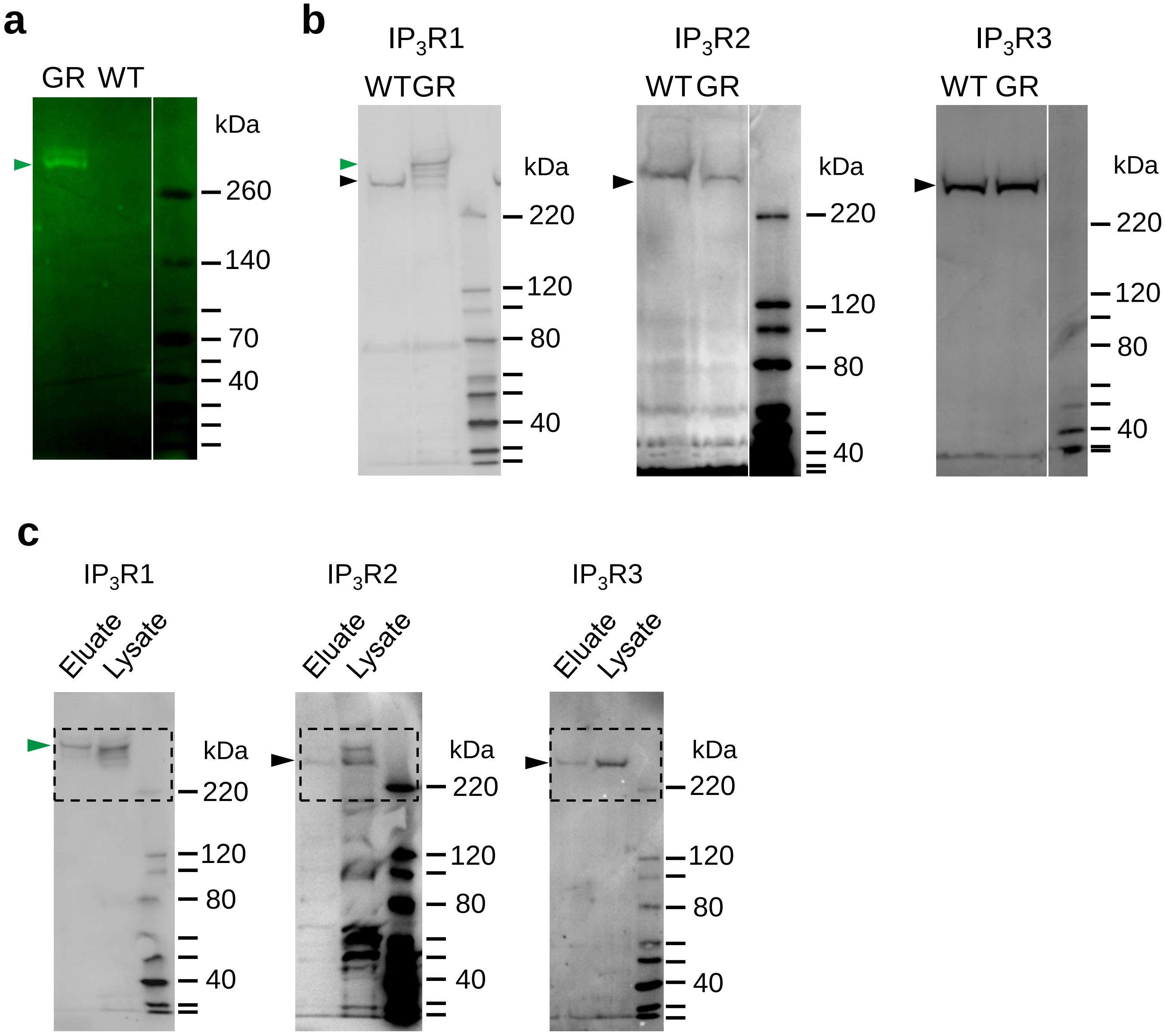


**Supplementary Figure 20 | Uncropped images of the gel and WBs shown in Figure 1.** (**a**) The gel shown in **Fig. 1a** is reproduced to show positions of $M_r$ markers (kDa: 260, 140, 100, 70, 50, 40, 35, 25, 15). (**b**) WBs from **Fig. 1c** shown with $M_r$ markers (kDa: 220, 120, 100, 80, 60, 50, 40, 30, 20). (**c**) WBs from **Fig. 1d** reproduced to show the entire WBs (dashed boxes show the regions presented in **Fig. 1d**). $M_r$ markers are also shown (kDa: 220, 120, 100, 80, 60, 50, 40, 30, 20).

**Supplementary references**


1. Frattini, A. *et al.* High variability of genomic instability and gene expression profiling in different HeLa clones. *Sci. Rep.* **5**, 15377 (2015).
2. Zhao, Y. *et al.* An expanded palette of genetically encoded $Ca^{2+}$ indicators. *Science* **333**, 1888-1891 (2011).
3. Ulbrich, M.H. & Isacoff, E.Y. Subunit counting in membrane-bound proteins. *Nat. Methods* **4**, 310-321 (2007).
4. Itzhak, D.N., Tyanova, S., Cox, J. & Borner, G.H. Global, quantitative and dynamic mapping of protein subcellular localization. *Elife* **5**, e16950 (2016).
5. Wagner, T., Kroll, A., Haramagatti, C.R., Lipinski, H.G. & Wiemann, M. Classification and segmentation of nanoparticle diffusion trajectories in cellular micro environments. *PLoS One* **12**, e0170165 (2017).
6. Chang, C.L. *et al.* Feedback regulation of receptor-induced $Ca^{2+}$ signaling mediated by E-Syt1 and Nir2 at endoplasmic reticulum-plasma membrane junctions. *Cell Reports* **5**, 813-825 (2013).
7. Alzayady, K.J. *et al.* Defining the stoichiometry of inositol 1,4,5-trisphosphate binding required to initiate $Ca^{2+}$ release. *Sci. Signal.* **9**, ra35 (2016).
