# Supplementary material for: Ca2+ signals initiate at immobile IP3 receptors adjacent to ER-plasma membrane junctions
Source: Nat Commun. 2017 Nov 15;8:1505. doi: 10.1038/s41467-017-01644-8 (PMC5686115; doi:10.1038/s41467-017-01644-8)
Supplement: Supplementary file 2 — Descriptions of Additional Supplementary Files [file 41467_2017_1644_MOESM2_ESM.docx]

**Descriptions of Additional Supplementary File**

File Name: Supplementary Movie 1

Descriptions: TIRFM images showing mobile and immobile puncta. TIRFM video (acquisition 10 fps, displayed at 15 fps) of a single EGFP-IP_3_R1 HeLa cell showing that many endogenous IP_3_Rs are mobile, while some are immobile; fps, frames per second. Time shown as h:min:s.ms.

File Name: Supplementary Movie 2

Descriptions: Mobile and immobile puncta do not interact. TIRFM video (acquisition 10 fps, displayed at 5 fps) of part of a cell showing a mobile IP3R punctum (centre of field) seemingly passing unperturbed through an immobile punctum. See also Fig. 3f.

File Name: Supplementary Movie 3

Descriptions: IP_3_Rs move rapidly within a less dynamic ER. TIRFM video (acquisition 0.8 fps, displayed at 10 fps) part of an EGFP-IP_3_R1 HeLa cell showing that IP_3_Rs (green) move rapidly within a less dynamic ER (mCherry-ER, red). Time shown as h:min:s.ms.

File Name: Supplementary Movie 4

Descriptions: EB3 is not associated with directionally moving IP_3_R puncta. TIRFM video (acquisition 0.7 fps, displayed at 20 fps) of an EGFP-IP_3_R1 HeLa cell expressing mCherry-EB3 showing that mobile IP_3_Rs (green) do not associate with EB3. Time shown as h:min:s.ms.

File Name: Supplementary Movie 5

Descriptions: Rapamycin-induced translocation of kinesin-1 moves IP_3_Rs. EGFP-IP_3_R1 HeLa cells were transfected with plasmids encoding KIF5c-tdTomato-FKBP and Lyn11-CFP-FRB and then treated with rapamycin (1 µM, added at 0.5 min) to induce dimerization of FKBP and FRB (Supplementary Fig. 13). The wide-field fluorescence images of the same cell (acquisition 14 frames/min, displayed at 15 fps) show accumulation of kinesin-1 (red, left panel) at the PM, where a sub-population of EGFP-IP_3_R1 also accumulates (green, right panel). Time shown as h:min:s.ms.

File Name: Supplementary Movie 6

Descriptions: Directionally moving IP_3_R puncta are unlikely to be held within vesicles. TIRFM video (acquisition 0.8 fps, displayed at 10 fps) of part of an EGFP-IP_3_R1 HeLa cell expressing mCherry-ER showing that mobile IP_3_R puncta (green) are not associated with mCherry-ER puncta.

File Name: Supplementary Movie 7

Descriptions: Histamine-evoked Ca^2+^ puffs. TIRFM video (acquisition 20 fps, displayed at 20 fps) of Ca^2+^ puffs evoked by histamine (10 µM, added at 1 s) in an EGFP-IP_3_R1 HeLa cell loaded with EGTA and Cal-590 showing multiple puff events that often revisit the same sites. Only the Cal-590 fluorescence is shown. Time shown as h:min:s.ms.

File Name: Supplementary Movie 8

Descriptions: Ca^2+^ puffs evoked by photolysis of caged-IP_3_. TIRFM video (acquisition 20 fps, displayed at 20 fps) shows Ca^2+^ puffs evoked by photolysis of caged-IP_3_ (flash at 2.5 s) in EGFP-IP_3_R1 HeLa cells loaded with EGTA and Cal-590 (left panel). Mobile (green and magenta) and immobile (white) IP_3_R puncta were identified by overlaying pseudocoloured images captured immediately before and after Ca^2+^ imaging (right panel, and superimposed on the left panel; see Supplementary Fig. 7). Ca^2+^ puffs (red) localize to immobile IP_3_Rs (white spots) and often revisit the same site. Time shown as h:min:s.ms.

File Name: Supplementary Movie 9

Descriptions: Expanding ER-PM junctions with mCherry-MAPPER displaces immobile IP_3_R puncta. TIRFM images (acquisition 0.2 fps, displayed at 1 fps) of an EGFP-IP_3_R1 HeLa cell expressing mCherry-MAPPER (red) shows massive expansion of the ER-PM junctions. Immobile IP_3_Rs are excluded from these exaggerated junctions, while mobile IP_3_Rs appear to pass through the junctional regions. Mobile (green and magenta) and immobile (white) IP_3_R puncta were identified by overlaying pseudocoloured images captured at 30-s intervals (right panel). Time shown as h:min:s.ms.
